# Genetic features and genomic targets of human KRAB-zinc finger proteins

**DOI:** 10.1101/gr.277722.123

**Published:** 2023-08

**Authors:** Jonas de Tribolet-Hardy, Christian W. Thorball, Romain Forey, Evarist Planet, Julien Duc, Alexandre Coudray, Bara Khubieh, Sandra Offner, Cyril Pulver, Jacques Fellay, Michael Imbeault, Priscilla Turelli, Didier Trono

**Affiliations:** 1 School of Life Sciences, Ecole Polytechnique Fédérale de Lausanne, 1015 Lausanne, Switzerland;; 2 Precision Medicine Unit, Lausanne University Hospital (CHUV) and University of Lausanne, 1010 Lausanne, Switzerland

## Abstract

Krüppel-associated box (KRAB) domain-containing zinc finger proteins (KZFPs) are one of the largest groups of transcription factors encoded by tetrapods, with 378 members in human alone. KZFP genes are often grouped in clusters reflecting amplification by gene and segment duplication since the gene family first emerged more than 400 million years ago. Previous work has revealed that many KZFPs recognize transposable element (TE)-embedded sequences as genomic targets, and that KZFPs facilitate the co-option of the regulatory potential of TEs for the benefit of the host. Here, we present a comprehensive survey of the genetic features and genomic targets of human KZFPs, notably completing past analyses by adding data on close to a hundred family members. General principles emerge from our study of the TE-KZFP regulatory system, which point to multipronged evolutionary mechanisms underlaid by highly complex and combinatorial modes of action with strong influences on human speciation.

Krüppel-associated box (KRAB) domain-containing zinc finger proteins (KZFPs) constitute one of the largest groups of transcription factors encoded by tetrapods, with 378 protein-coding representatives in human alone (Supplemental Table S1). KZFPs are characterized by an N-terminal KRAB domain and a C-terminal array of zinc fingers (ZFs) conferring sequence-specific polynucleotide binding potential. Sequence specificity is conferred by three amino acids within each ZF interacting with a triplet of bases in the polynucleotide target, with a minor contribution from a fourth ZF residue (Elrod-Erickson et al. 1998). The juxtaposition of the DNA-contacting amino acids of each ZF within the zinc finger array of a KZFP is designated as its zinc fingerprint. KZFP genes are often grouped in clusters reflecting their amplification by gene and segment duplication since the family first emerged more than 400 million years ago in the last common ancestor of lungfish, coelacanth, and tetrapods (Huntley et al. 2006; Nowick and Stubbs 2010; Imbeault et al. 2017). The KRAB domain of all evolutionarily recent human KZFPs recruits tripartite motif containing 28 (TRIM28), also known as KRAB-associated protein 1 (KAP1), which acts as a scaffold for a heterochromatin-inducing complex, repressing transcription over KZFP-bound loci and flanking regions (Urrutia 2003). Older, more conserved KZFPs often harbor variant KRAB domains that display functionally diverse TRIM28-devoid protein interactomes (Helleboid et al. 2019). Cumulated work has identified transposable elements (TEs) as major targets of KZFPs, which likely evolved both to control the spread of these genetic invaders and to facilitate the domestication of their regulatory potential (Wolf and Goff 2009; Jacobs et al. 2014; Najafabadi et al. 2015; Schmitges et al. 2016; Imbeault et al. 2017; Bruno et al. 2019). TE-derived sequences make up a readily recognizable 50% of the human genomic DNA, a likely underestimation of their real contribution to our genetic makeup as their signature features get lost over time because of genetic drift. Most human TEs are retrotransposons spreading by a copy-and-paste mechanism, be they LTR (long terminal repeat)-containing endogenous retroviruses (ERVs), long and short interspersed nuclear elements (LINEs and SINEs), or the composite SINE-variable number of tandem repeats (VNTR)-*Alu* (SVAs). ERVs and LINEs encode the reverse transcriptase and endonuclease activities necessary for their retrotransposition, whereas the nonautonomous SINE and SVA elements rely on LINE proteins for spreading. Due respectively to internal recombination and abortive retrotranscription, incomplete ERV and LINE integrants abound, for the former as solo-LTRs and for the latter as 5′-truncated units of various lengths (Kojima 2018).

Uncontrolled TE activity is deleterious to an organism notably because new insertions can disrupt the genome and cause disease (Hancks and Kazazian 2016; Kim et al. 2016; Durnaoglu et al. 2021). Accordingly, TEs are tamed by several general mechanisms, whether protein-based repressors such as the KZFP/TRIM28 or the HUSH complexes (Seczynska et al. 2022) or RNA-based mechanisms such as piRNAs (Ozata et al. 2019), the latter playing a prominent role to control TEs during the genome reprogramming associated with gametogenesis. However, cumulated evidence indicates that KZFPs do more than simply preventing transposition, be it only because most TE integrants remain targeted by these proteins many millions of years after they have lost all replicative potential because of mutations (Imbeault et al. 2017). This has led to the suggestion that KZFPs act to facilitate the domestication of the regulatory potential of TEs, which had been proposed long ago to be key to the establishment and evolution of gene regulatory networks (Britten and Davidson 1969; Trono 2015). In line with this hypothesis, individual human KZFPs have been found to be implicated in a variety of biological processes, including embryonic genome activation, gastrulation, gametogenesis, imprinting, placentation, brain development, adipogenesis, and angiogenesis (Wagner et al. 2000; Hayashi and Matsui 2006; Li et al. 2008; Quenneville et al. 2011; Zeng et al. 2012; Yang et al. 2017a; Chen et al. 2019; Pontis et al. 2019, 2022; Takahashi et al. 2019; Playfoot et al. 2021; Iouranova et al. 2022; Wang et al. 2022). An important step towards defining the roles of all human KZFPs lies in a more complete characterization of their genomic targets. Even though comparisons of zinc fingerprints across KZFPs have been successfully used to establish evolutionary relationships between proteins (Imbeault et al. 2017), complex interactions between ZFs in an array (Wolfe et al. 2001) make in silico predictions of ZF binding extremely challenging, thus targets still require to be identified experimentally. Previous studies have provided significant advances in this direction, collectively delineating the binding preferences of some 240 KZFPs (Najafabadi et al. 2015; Imbeault et al. 2017; Helleboid et al. 2019; Iouranova et al. 2022). Here we have contributed to this effort by unveiling genomic target site locations bound by 94 previously uncharacterized human KZFPs. With these and data previously obtained by our and other groups, the genomic targets of about 95% human KZFPs have now been identified, which together with an examination of genetic features of this gene family allows one to delineate some interesting general principles.

## Results

### Genomic distribution and evolutionary features of human KZFP genes

**Figure 1.**
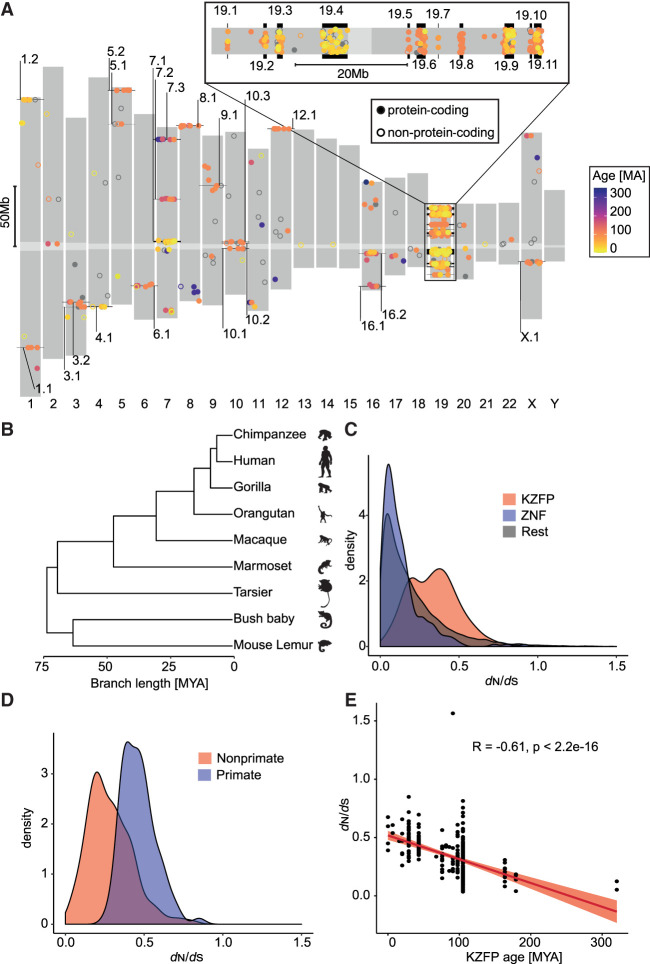
Human KRAB-zinc finger proteins (KZFPs) and their evolution in the primate lineage. (*A*) Dots indicate relative chromosomal position of KZFP genes (defined by juxtaposed KRAB- and zinc finger-coding domains), with the color code indicative of age (gray for unassigned) and numbered clusters pointed to in black. Hollow circles indicate non-protein-coding genes. A higher magnification of Chromosome 19 is presented on *top*. Centromeres are indicated in light gray. (*B*) Phylogenetic tree of primate species used to calculate natural selection of human genes, with branch length indicating approximate time of divergence in million years (MYA). Silhouettes courtesy of PhyloPic (http://phylopic.org/). (*C*) Distribution of PAML *d*_N_/*d*_S_ values of natural selection for KZFPs (red), nonKRAB ZFPs (blue), and all remaining genes in the genome (gray). (*D*) *d*_N_/*d*_S_ distribution of primate-specific KZFPs (blue) and older (red) KZFP genes. (*E*) Spearman's correlation of the *d*_N_/*d*_S_ values and estimated age of KZFP genes. The linear regression and 95% confidence interval are shown in red.

As a starting point, we updated the census of human KZFP genes, using hg19 as data source. We identified 467 pairs of neighboring sequences corresponding to KRAB and C2H2 poly-zinc finger domains, 378 of which were predicted to encode full-length KZFPs. We could also delineate 31 clusters, that is, groups of at least three genes separated by <250 kb as previously defined (Huntley et al. 2006) (Fig. 1A; Supplemental Table S1). Eleven of these clusters reside on Chromosome 19, collectively hosting 246 KZFPs (219 of which are protein coding). Using previously estimated evolutionary ages (Imbeault et al. 2017), we further determined that isolated protein-coding KZFP genes tend to be older than their cluster-associated counterparts (*P* = 3.5·10^−6^, Wilcoxon rank-sum test [WRS]). This fits with the proposal that Chromosome 19 is the main region of emergence of new KZFP genes (Lukic et al. 2014) and suggests that escaping the tumultuous environment of this chromosome facilitated the fixation of older family members. However, this is not a strict rule, as the long arm of Chromosome 7 hosts both a cluster of some of the most ancient KZFPs (*ZNF282*, *ZNF777*, and *ZNF783*) near its distal end as previously noted (Liu et al. 2014), and a cluster of primate-specific KZFPs (*AC115220.1*, *ZNF727*, *ZNF735*, *ZNF679*, *ZNF736*, *ZNF680*, *ZNF107*, *ZNF138*, *ZNF273*, and *ZNF117*) near the centromere (Fig. 1A; Supplemental Fig. S1).

To complement this initial analysis, we examined the recent evolution of KZFP genes in the primate lineage as previously described (Takahashi et al. 2019). For this, we determined their gene-wide ratio of nonsynonymous (missense) (*d*_N_) to synonymous (*d*_S_) substitutions (*d*_N_/*d*_S_), based on genome sequence data from human, chimpanzee, gorilla, orangutan, macaque, marmoset, tarsier, galago (a.k.a. bush baby), and mouse lemur, that is, over ∼6 to ∼74 million years of divergence (Fig. 1B). We found KZFPs to have significantly higher *d*_N_/*d*_S_ values than genes coding for other proteins (*P* = 1.46·10^−49^, WRS), including KRAB-less ZF proteins (*P* = 3.04·10^−46^, WRS) (Fig. 1C), confirming previous observations that KZFPs are a rapidly evolving gene family (Emerson and Thomas 2009; Najafabadi et al. 2017). We also noted that the distribution of *d*_N_/*d*_S_ values was bimodal among KZFP genes, with younger, primate-specific family members displaying higher scores than evolutionarily older ones (*P* = 2.69·10^−21^, WRS) (Fig. 1D) and with the *d*_N_/*d*_S_ ratio of KZFP genes anticorrelated with their estimated age (rho = −0.61) (Fig. 1E). This slowdown in the rate of evolution of older KZFPs, which is a prerequisite for their conservation, indicates different evolutionary pressures at work in the KZFP gene family.

### Conservation-related gradient of human KZFP gene polymorphisms

**Figure 2.**
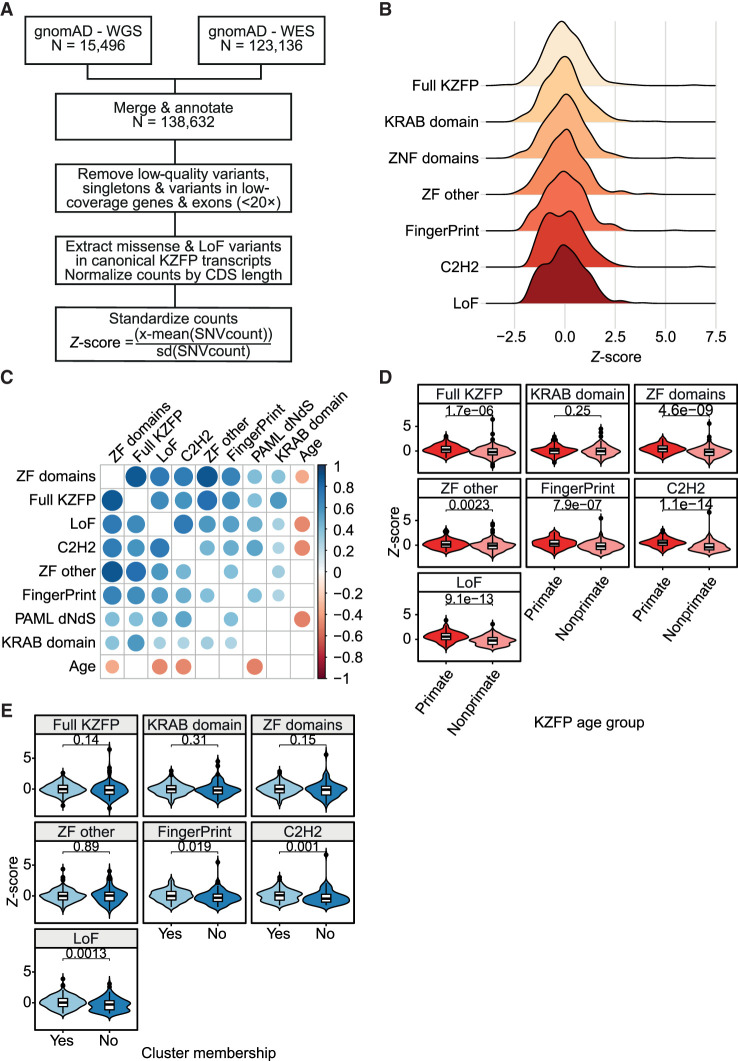
Coding constraints of KZFP genes. (*A*) Schematic of genetic constraint Z-score calculation. WGS/WES, whole genome/exome sequencing; LoF, loss-of-function variant; CDS, coding sequence. (*B*) Distribution of indicated *Z* scores; a lower score indicates increased constraint compared to the average of all KZFPs. Full KZFP, all variants within the canonical KZFP transcript; KRAB domain, only variants in the KRAB domain; ZF domains, variants within the ZF domains; ZF other, variants in nonfunctional positions within the ZF domains; FingerPrint, variants in the ZF fingerprint positions; C2H2, variants in the cysteine or histidine positions of the ZF domains; LoF, loss-of-function variants. (*C*) Correlation plot showing the Spearman's correlations between: the *Z* scores defined in *B*, level of natural selection (PAML *d*_N_/*d*_S_), and estimated age of KZFPs. The colors and their intensity represent the direction and strength of the correlations, with blue representing a positive and red a negative correlation. Only significant correlations after Bonferroni correction are shown. (*D*) Primate versus nonprimate KZFP constraint across indicated KZFP domains or residues. (*E*) Relative constraint of indicated regions for KZFPs inside versus outside clusters. *P*-values were calculated using the Wilcoxon rank-sum test (WRS).

To further investigate this question, we observed the coding constraint of KZFP genes in the human population. The coding constraint of a gene or fragment thereof reflects the strength of selective pressures imposed on its sequence, hence is linked to the relative functional importance of the corresponding protein or protein domain for a given species. Typically, highly constrained coding regions correspond to loci whereas mutations are either associated with disease or are completely absent because they cause sterility or embryonic lethality. To calculate the coding constraints imposed on human KZFP genes, we examined genetic variation among 138,632 individuals (15,496 genomes and 123,136 exomes) cataloged in gnomAD v.2.0.2 (https://gnomad.broadinstitute.org/). After removing coding sequences with low coverage and dismissing singletons to reduce the impact of false positives resulting from sequencing or alignment errors, we extracted protein-altering variants (missense and predicted loss of function [LoF] by frameshift, gain of stop codon, or alteration of essential splice sites) within the canonical transcripts of all remaining KZFPs (n = 361). For the estimation of gene-wide constraint, we normalized the number of variants for the length of the canonical coding sequence and translated the result into a *Z* score to standardize values (Fig. 2A,B). Accordingly, negative deviation from the mean is a sign of increased purifying selection as a consequence of reduced frequency of protein-altering variants. However, we did not correct for a theoretically expected number of mutations as frequently performed in this type of analysis because the unstable structure of the ZF array-coding region of KZFP genes renders this parameter unpredictable (Yang et al. 2017b). Gene-wide, LoF, and ZF domain-specific scores modestly correlated with previously measured *d*_N_/*d*_S_ ratios and with the age of the KZFPs (Fig. 2C). Examining individual domains revealed that this association stemmed mainly from the ZF C2H2- and to a lesser extent fingerprint-coding sequences. Of note, other codons of the ZF-coding regions displayed no significant constraint, confirming that essential positions in ZFs are limited to the structure-conferring cysteine and histidine residues and the target-defining fingerprint residues at positions −1, +3, and +6 of the ZF alpha helix (Najafabadi et al. 2017). Primate-restricted, younger KZFPs were significantly less constrained both in terms of LoF (*P* = 9.1·10^−13^, WRS) and missense variation (*P* = 1.7·10^−6^, WRS) than their older counterparts (Fig. 2D), with the difference mainly residing in sequences coding the poly-ZF (*P* = 4.6·10^−9^, WRS) rather than the KRAB domain (*P* = 0.25, WRS). Within ZFs, the C2H2- and fingerprint-defining positions were again the most influential (*P*_ZFc2h2_ = 1.1·10^−14^ and *P*_ZFprint_ = 7.9·10^−7^, WRS), compared to the other nonfunctional positions of the ZF domains (*P*_ZF other_ = 0.002, WRS). Correlating with their age, isolated KZFP genes were more constrained at sequences encoding the ZF C2H2 residues (*P* = 0.001, WRS) and fingerprint-defining positions (*P* = 0.02, WRS). Furthermore, they displayed lower LoF scores (*P* = 0.001, WRS) than their cluster-associated counterparts, consistent with their stabilization over longer evolutionary times (Fig. 2E). However, coding constraints were also highly heterogeneous within most clusters, indicating that differential selective pressures are rapidly exerted on members of the same gene cluster (Supplemental Fig. S2).

When looking at the most conserved KZFPs, no LoF variants were detected among all examined individuals for *ZFP92*, *ZNF606*, *ZNF81*, *ZNF777*, *ZNF250*, and *ZNF597*, which all are 105 myo (million years old) except *ZNF777* which is 312 myo. The *ZFP92*, *ZNF81*, and *ZNF777* genes were also devoid of any missense mutations in their C2H2- or fingerprint-coding positions, whereas some were detected in *ZNF250*, *ZNF597*, and *ZNF606* albeit at extremely low allele frequencies. For a majority of other KZFPs (n = 213), some heterozygous but no homozygous LoF variants were observed. Nevertheless, a significant number (n = 148) presented homozygous LoF variants in at least two individuals, suggesting reduced constraint (*P* < 2.22·10^−16^, WRS) (Supplemental Fig. S2). On average, members of this subgroup had a younger estimated age than the rest of the KZFPs (*P* = 2.9·10^−13^, WRS), showing that KZFPs that are more conserved during evolution also have higher constraint in the human population, delineating them from younger faster evolving family members.

### Differential coding constraints of human KZFP paralogs

**Figure 3.**
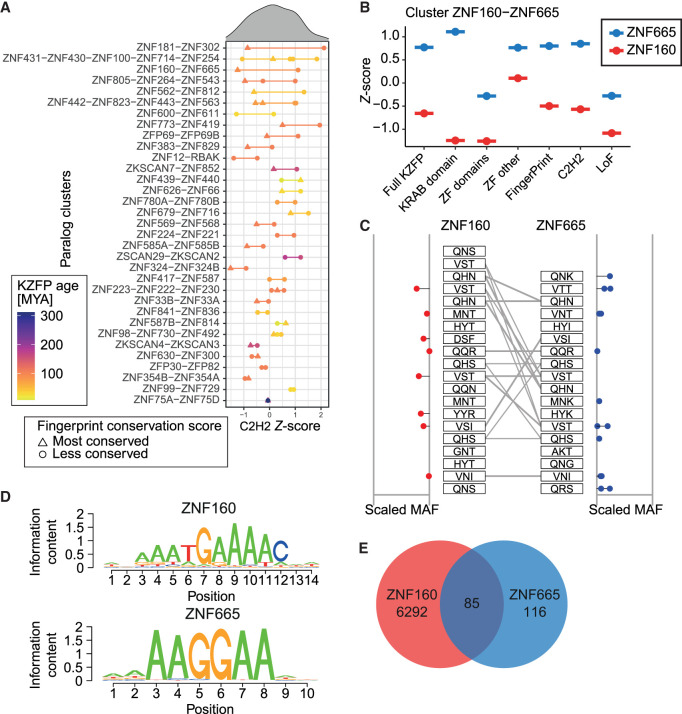
Coding constraints of KZFP paralogs. (*A*) Distribution of C2H2 constraint *Z* scores for indicated sets of KZFP paralogs, arranged from *top* to *bottom* according to difference within pairs. Each KZFP is colored according to their respective age, with the line separating them colored as the mean age of the pair. The paralog within each pair with the most conserved fingerprint across evolutionary time is marked by a triangle, whereas less or identically conserved KZFPs are marked by a dot. The order of the *y*-axis labels corresponds to the order of the colored points on the graph. (*B*) Differential constraint *Z* scores for indicated domains of paralogs ZNF160 and ZNF665. (*C*) Zinc fingerprints of ZNF160 and ZNF665 with the scaled minor allele frequency (MAF) of identified missense variants indicated on the sides. Gray lines indicate identical zinc fingerprints. (*D*) Consensus DNA binding motifs of ZNF160 and ZNF665. (*E*) Venn diagram of ChIP-exo peaks of ZNF160 and ZNF665 in HEK293T cells.

To further investigate the connection between the coding constraints and the evolutionary history of KZFP genes, we examined 33 sets of KZFP paralogs, identified based on similarities between their zinc fingerprints (Imbeault et al. 2017). For 28 of them, both members of a paralog pair were located within the same chromosomal cluster. Significant differences in their coding constraint were noted, especially at the C2H2-coding positions, with some pairs of paralogs displaying closely similar coding constraints (e.g., *ZNF75A* and *ZNF75D*) whereas others were markedly divergent (e.g., *ZNF160* and *ZNF665*) (Fig. 3A). The level of divergence was not related to the age of the paralog pairs (*P* = 0.21, WRS). However, the more constrained paralog within a pair was usually also the most conserved in evolution (Fig. 3A). For instance, the ∼90 myo *ZNF160* was markedly more constrained than its ∼29 myo *ZNF665* paralog, both at C2H2-coding positions and across other features (Fig. 3B). A closer examination of ZNF160 and ZNF665 zinc fingerprints revealed that some ZFs were completely constrained in both KZFPs, whereas others were more flexible (Fig. 3C). ChIP-seq analyses confirmed that these proteins recognized closely related sequence motifs (Fig. 3D) in overlapping sets of genomic targets (Fig. 3E), notably some LINE-1 integrants (Supplemental Fig. S3A). Furthermore, the two paralogs were noted to have roughly similar expression patterns across 40 tissues according to the GTEx database (GTEx Consortium 2017) (rho = 0.89) (Supplemental Fig. S3B). This is contrasted with the more global observation that more constrained KZFPs were generally expressed at higher levels than their more flexible counterparts (Supplemental Fig. S3C), in line with previous reports (Lek et al. 2016). Together these results illustrate how the previously shown divergence between rapidly evolving and more conserved KZFPs can arise in pairs of paralog KZFP genes.

### The repertoire of human KZFP genomic targets is strongly biased towards TEs

**Figure 4.**
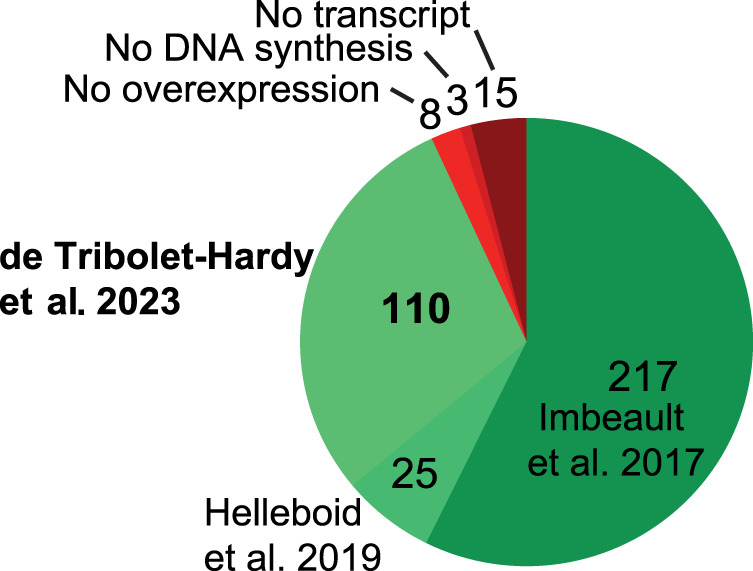
Profiling of the human KZFPs. Pie chart of the data on all 378 protein-coding KZFPs. “No overexpression” indicates the number of KZFPs where the codon-optimized construct did not yield sufficient protein. “No transcript” represents KZFPs with no annotated transcript containing both the KRAB and zinc finger domains simultaneously. “No DNA synthesis” indicates the number of KZFP CDSs that could not be synthesized, with a minimum of two tries. “de Tribolet-Hardy et al. 2023” refers to the present work.

Following this, a deeper comprehension of KZFP target sequences was imperative to enhance our understanding of the consequences and factors influencing KZFP evolution. We previously identified the genomic targets of 242 human KZFPs through chromatin immunoprecipitation followed by DNA sequencing (ChIP-seq) (Imbeault et al. 2017; Helleboid et al. 2019). Here we extended these analyses to an additional 94 family members, similarly using HA-tagged derivatives overexpressed in 293T cells transduced with dox-inducible lentiviral vectors (Imbeault et al. 2017). Of the remaining 26 KZFPs, DNA could not be successfully synthesized in three cases, whereas transduction yielded no or little protein in another 23 (Fig. 4; Supplemental Table S2). This large consistent data set allowed us to reduce both the number of nonspecific and total peaks in our experiments by removing sequences present in all ChIPs irrespective of the bait protein (Supplemental Fig. S4A,B). As a consequence, we considered enrichment over specific DNA sequences as the main criterion for assessing the quality of a ChIP, rather than the absolute peak number (see Supplemental Fig. S4C for an example). Of note, we retained for the present report nine KZFPs for which the ChIP did not give any peak and 24 that yielded fewer than 10 peaks, provided that these KZFPs were robustly expressed, and the number of sequencing reads were similar as those obtained for other KZFPs in the same batch and in other experiments. We did so, on the one hand, because some KZFPs might not interact with DNA, at least in the cell system used here, and, on the other hand, because certain ChIPs with very low peak numbers still exhibited enrichments at specific locations (see Supplemental Fig. S4D for an example). We fully reckon that additional testing will be required to confirm the affinity of these KZFPs for specific DNA sequences, or lack thereof, including by performing analyses in cells where they are physiologically expressed. Altogether, integrating our cumulated data with those previously obtained by other groups (Frietze et al. 2010; The ENCODE Project Consortium 2012; Yan et al. 2013; Schmitges et al. 2016; Venkataraman et al. 2018; Partridge et al. 2020; Haring et al. 2021), we could build a lexicon constituted by the genomic targets of 358 human KZFPs, including replicates for 78 of them (Supplemental Table S2). These data are displayed on KRABopedia (https://tronoapps.epfl.ch/web/krabopedia/), where analyses of replicates (as in Supplemental Fig. S4B,C) can also be found, together with information on the age, the genomic location, expression patterns, and, when available, the protein interactome of all human KZFPs examined to date.

**Figure 5.**
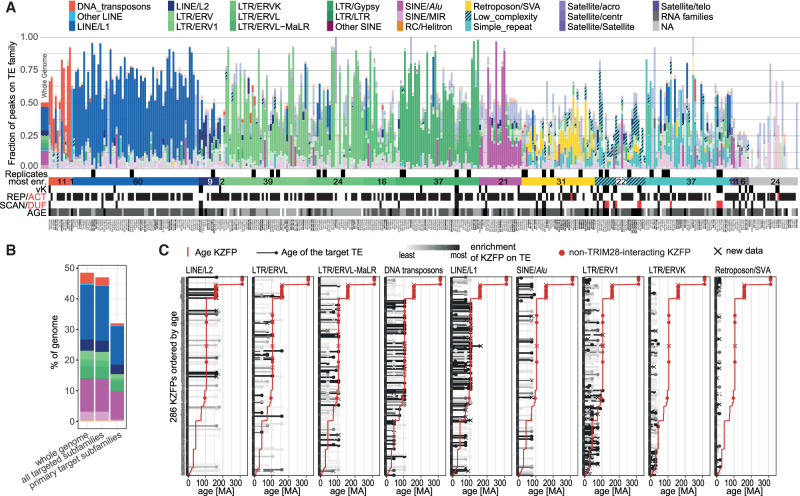
Targets of human KZFPs. (*A*) Bar graph showing the fraction of peaks over repetitive element (RE) families for all our conducted experiments (*x*-axis) (external data are shown in Supplemental Fig. S5), ordered by the most enriched family, indicated by the horizontal bar *below* along with the number of KZFPs for each category. Significant enrichments (FDR > 0.05) are shown in fully opaque colors whereas nonsignificant enrichments are transparent. The *leftmost* bar shows the percentage of the genome occupied by each RE family. Replicate experiments are indicated by black squares *above* the horizontal bar. Different aspects of each KZFP are shown *below* the horizontal bar: vK = variant KRAB according to Helleboid et al. (2019), REP/ACT = repressor and activator KZFPs according to Tycko et al. (2020), SCAN/DUF = KZFP carrying an additional SCAN or DUF3669 domain. Age: Black = >105 myo, dark gray = >105 myo years (placental mammals), light gray = > 74 myo years (primates), white = no data. The total number of peaks per experiment is indicated in brackets after the KZFP name *below* each bar. Names of KZFPs with new data are shown in saturated black; previously published KZFPs are shown in gray. (*B*) Bar graph showing the genome occupancy of targeted TE subfamilies. The *left* stack of bars shows the fractions of the genome covered by TEs, the *central* stack shows the coverage by all TE subfamilies which are targeted by a KZFP (FDR > 0.05), and the *right* stack shows the coverage of the TE subfamilies which are the primary target of one or more KZFPs (10% highest −log_10_[FDR]). Bars are colored according to the TE families to which the subfamilies belong, with the same color code as in *A*. (*C*) Age of KZFP and their target TEs. KZFPs (rows) are ordered by age, shown as a red line. Their targets are split into different subplots by family (excluding families targeted by <20 KZFPs) and their age is shown as black or gray bars with a dot on *top*. The gray level of the TE targets shows the level of enrichment of the given KZFP for the subfamily with black showing the target with the highest −log_10_(FDR) linearly scaling to 0 (white). If the KZFP is enriched on several subfamilies of the same family, the lowest FDR is shown. Red dots indicate KZFPs which are unlikely to interact with TRIM28 as defined by Helleboid et al. (2019). KZFPs with new data presented in this study are marked by a cross.

As previously observed through studies on smaller subsets of KZFPs (Najafabadi et al. 2015; Imbeault et al. 2017; Helleboid et al. 2019), our integrated analysis confirmed that the vast majority of human KZFPs are foremost enriched at TE-derived loci (Fig. 5A; Supplemental Fig. S5). We attribute the generally lower fraction of peaks on TEs in external data sets (Supplemental Fig. S5) to the above-mentioned improved filtering of peaks we could apply on the data presented in Figure 5A. Because of the close relationship between different TE subfamilies and to the production of enrichments rather than binary results by the ChIP-seq technique, we generally saw several TE subfamilies significantly enriched with a given KZFP bait. However, in most cases a few subfamilies stood out as much more enriched than others. We defined the identified sequences as primary targets of the ChIP'ed KZFPs if within an arbitrary cutoff (10% of the log_10_ of the lowest false detection rate [FDR]) and designated the remainder as potential secondary targets (Supplemental Table S3; Supplemental Fig. S6). Of 349 KZFPs with clearly identifiable targets, 120 were preferentially enriched at ERVs, 70 at LINEs, 21 at SINEs/*Alus*, 32 significantly bound SVAs, and 11 were rather found at DNA transposons. The remaining 71 KZFPs mapped to a mixture of low complexity or simple repeats, satellite DNAs and tRNAs. For 24, no particular class of genomic entity could be singled out perhaps due in part to difficulties in sequences alignment, notably in telomeric and centromeric regions (Fig. 5A; Supplemental Fig. S5 with further details on https://tronoapps.epfl.ch/web/krabopedia/). Altogether, about two-thirds of known human TE subfamilies were found to constitute primary targets of at least one KZFP, this number exceeding 95% if secondary targets were also considered (Fig. 5B). Thus, our data indicate that both a large majority of KZFPs bind TEs and a large majority of TEs are bound by KZFPs, further strengthening the evolutionary and functional link between these two genetic entities. Confirming with this larger data set a trend noted previously (Imbeault et al. 2017), the evolutionary times of TEs and of their controlling KZFPs most often coincided (Fig. 5C). When comparing the age of KZFPs (red line) with the age of their targets (black bars), contemporary waves of KZFPs and TEs emergence can be observed. For example, LINE-1 subsets and ERVL that emerged some 105 million years ago were predominantly bound by KZFPs of similar ages, an observation that held true for the younger ERV1 and ERVK and their cognate ligands (see Supplemental Table S4). However, such evolutionary pairing did not apply to SINE and SVA elements, both of which were found to be targeted primarily by older KZFPs, including family members not interacting with TRIM28 (red dots). Another interesting case are LINE-2 elements, half of which were targeted by contemporary KZFPs whereas the other half was bound by evolutionarily younger ligands.

### TEs are avid KZFP recruiters

**Figure 6.**
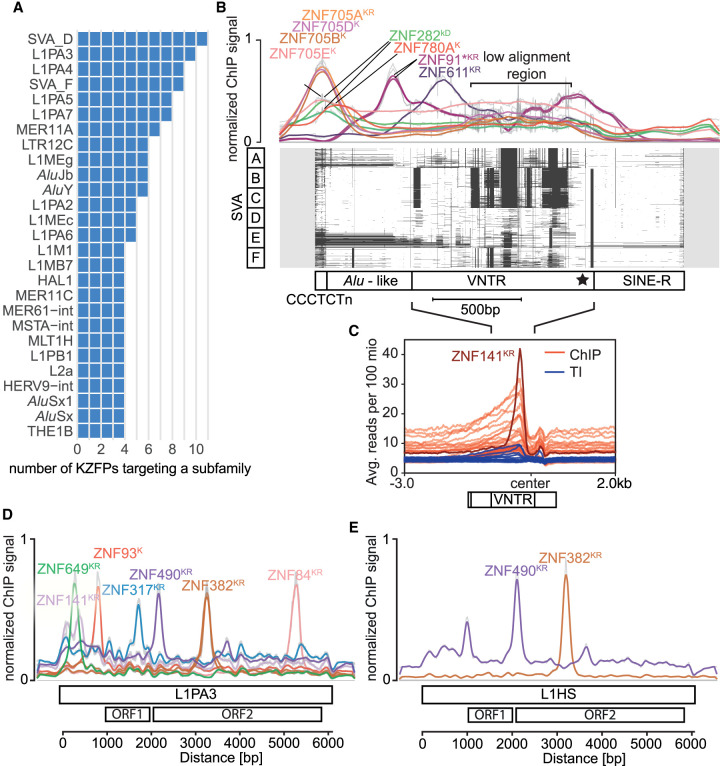
TE families are targeted by multiple KZFPs. (*A*) Bar graphs showing the TE subfamilies targeted by the largest number of different KZFPs. Only KZFPs targeting the subfamily as their primary targets were considered (−log_10_(FDR) within 10% of the highest ­−log_10_(FDR) for that KZFP). (*B*) KZFP signal over the multiple sequence alignment (MSA) of SVA subfamilies A to F. *Top*: Line graph of the normalized cumulative reads for each position from the indicated ChIP-seq and -exo experiments. External data sets are marked with stars. *Bottom*: MSA plot of 100 of the longest SVA sequences for each subfamily indicated on the *left*, 200 bp of nonaligned extensions are added around elements shown in gray, white depicts aligned regions, and black gaps in the alignment. For visibility, places in the alignment (columns) with more than 85% gaps were removed. The approximate different domains of the SVAs are indicated *below*, adapted from Hancks and Kazazian (2010); the star indicates the center region for *C*. (*C*) Signal over the low alignment region of the remaining SVA binders centered on the 3′ end of the VNTR (without alignment of sequences). ChIP signals for KZFPs enriched on SVAs are shown in red (ZFP57, ZFP92*, ZNF14*, ZNF141, ZNF155*, ZNF215, ZNF25, ZNF256, ZNF263, ZNF268*, ZNF28, ZNF30, ZNF41*, ZNF415*, ZNF461*, ZNF500*, ZNF556*, ZNF560*, ZNF57*, ZNF587B*, ZNF597, ZNF624*, ZNF641, ZNF689*, ZNF699*, ZNF747*, ZNF813*, ZNF852*, and ZNF878*; * = new data in this publication) with the signal for ZNF141 shown in dark red. Input signals for the presented ChIPs are shown in blue. (*D*,*E*) Binding sites of KZFPs on L1PA3 and L1HS elements. Elements were aligned the same way as in *A* and the normalized ChIP-seq and -exo signals are shown for each aligned position. External data sets are marked with stars. K = standard KRAB, k = variant KRAB, D = DUF domain, R = repressor; according to Helleboid et al. (2019) and Tycko et al. (2020). (*D*) 1000 L1PA3 elements were aligned. (*E*) 382 full-length L1HS elements were aligned. Multimapped reads were included for the signals in panels *B*–*E*.

Our data confirm that most TE subfamilies are bound by more than one KZFP (Fig. 6A; Supplemental Fig. S7A). This feature is particularly striking for SVAs, considering the relatively young age of this class of retrotransposons (around 15 million years for the oldest SVA-A), their small size (on average 2500 bp), and the low number of their integrants (some 3500 for the entire family) (Kojima 2018; Wang et al. 2005). SVAs are nonautonomous composite elements made up by the juxtaposition of an *Alu*-like sequence, a VNTR, and an ERV-derived 3′ region called SINE-R, somewhat of a misnomer because it is not related to the SINE family of TEs (Ono et al. 1987). A few tens of SVAs (belonging to the youngest, human-specific SVA-F subset) are still transposition-competent, using for their replication the reverse transcriptase and endonuclease activities provided by LINE-1 in *trans*. Many SVAs, notably from the SVA-D subgroup, provide enhancers active in early embryogenesis and/or in adult tissues, including at KZFP gene clusters (Gianfrancesco et al. 2019; Pontis et al. 2019, 2022; Haring et al. 2021). The distribution of KZFP binding sites over SVA sequences revealed three distinct patterns (Fig. 6B). ZNF705A, B, D, and E as well as ZNF282 and ZNF780A were enriched over the *Alu*-like segment, whereas the previously described (Jacobs et al. 2014) ZNF611 and ZNF91 were found to bind to the 5′ end of the VNTR. However, the vast majority of ChIP signals overlapped with the more distal, highly variable part of the VNTR, in which 29 KZFPs were enriched. A closer look at ZNF141, which yielded the strongest signal (Fig. 6C), revealed that ZNF141 was also enriched over L1PA3 and L1PA2 integrants and SATR1 Satellite repeats (Supplemental Fig. S7B), and that the same previously identified binding motif (Weirauch et al. 2014) was found in all three types of TE targets, confirming *bona fide* affinity for this region of SVAs (Supplemental Fig. S7C). LINE-1 integrants, at least when full-length, were also targeted by multiple KZFPs, some binding towards the 5′ end of these integrants, hence likely to repress their transcription, and others recognizing downstream regions dispersed all the way to their 3′ end (Fig. 6D). Furthermore, comparing the KZFP recruitment patterns of recent and older LINE-1 subfamilies pointed to the influence of differential evolutionary forces. For instance, the TRIM28-binding ZNF93 repressor, which emerged in the last common ancestor of apes and Old World monkeys, recognizes the promoter regions of ∼27 myo L1PA6 to ∼16 myo L1PA3 but not that of ∼3 myo L1HS (Jacobs et al. 2014), whereas the more distally binding ZNF382 (Imbeault et al. 2017) and ZNF490, both also TRIM28 recruiters but ∼105 myo (Helleboid et al. 2019), bind integrants from all of these LINE-1 subsets (Fig. 6D,E).

### Multipronged modes of evolution of the TE-KZFP interaction

**Figure 7.**
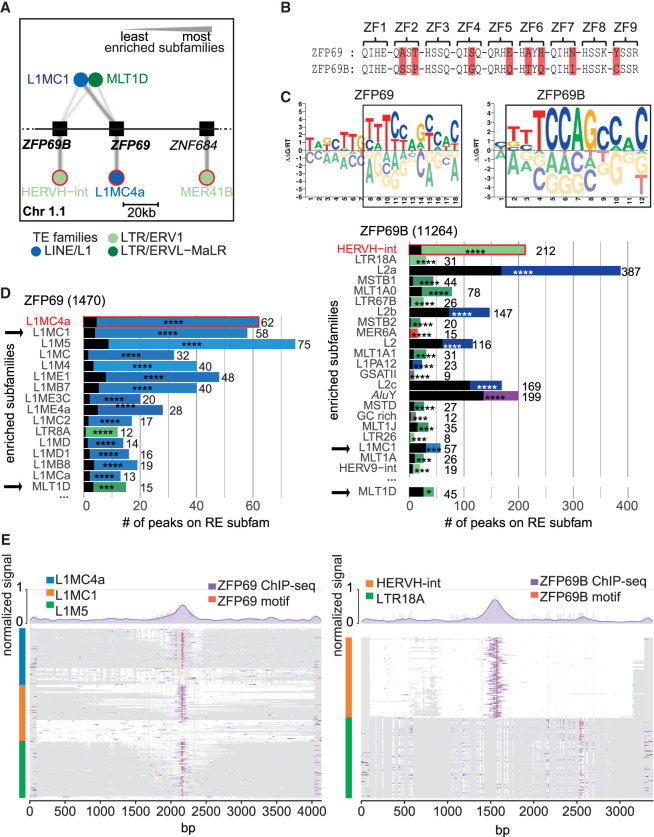
Evolution of TE-KZFP interaction. (*A*) Network for cluster Chr 1.1 in which targets (circles) of each KZFP (squares) are shown as connected edges and the amount of binding is represented by the line thickness. The thickest line for each KZFP represents the TE subfamily with the highest −log_10_(FDR) and then scales linearly to the lowest value. For visibility, only the best targets (*below*) and shared targets (*above*) are shown. The TE subfamilies are colored according to their families. Primary targets for each KZFP are highlighted in red. (*B*) Zinc fingerprints of ZFP69 and ZFP69B. The DNA-contacting amino acids for zinc finger (ZF) are shown; differences are highlighted in red. (*C*) DNA binding motifs of ZFP69 and ZFP69B as identified by Weirauch et al. (2014). Regions of high similarity are framed by a black square. (*D*) Enrichment of peaks over different repetitive element subfamilies. Subfamilies with FDR > 0.01 are shown. The width of the colored bars represents the number of peaks per subfamily also shown as a number on the *right* of the bar. The black transparent bars represent the expected number of peaks following a random distribution. The FDR of the enrichment is shown with stars (FDR > 0.0001 = ****, >0.001 = ***, >0.01 = **, >0.05 = *, ≥0.05 = n.s.). Rows are ordered by FDR. The number next to the title indicates the total number of peaks for the experiment. Primary targets for each KZFP are highlighted in red and shared subfamilies between the two panels are indicated by black arrows. (*E*) MSA over the three most enriched targets of ZFP69 (*left*) and ZFP69B (*right*). Up to 200 elements for the indicated targets (blue, orange, and green) were aligned, selecting first elements overlapping with a peak and then the longest elements. White regions in the plots indicate aligned sequences; gray regions indicate gaps. The signal of ZFP69 and ZFP69B ChIPs was laid over their respective alignments in purple. The locations of their motifs from panel *C* are shown in red. The normalized signal can be seen as a line plot *above* the MSA plot.

KZFPs present within the same cluster are often related in sequence because in many cases they are derived from each other (Lukic et al. 2014). For example, *ZFP69* and *ZFP69B*, encoded side-by-side in the Chr 1.1 cluster (Fig. 7A), are both ∼105 myo, having emerged in the last common ancestor of primates and armadillo (Supplemental Fig. S8A). Both ZFP69 and ZFP69B display significant and conserved similarities in their zinc fingerprints (Fig. 7B; Supplemental Fig. S8A) and DNA binding motives (Fig. 7C). This may explain why only one of the two paralogs was retained in many rodents and even-toed ungulates (Supplemental Fig. S8A). However, their primary targets differ, with human ZFP69 preferentially recognizing a mammalian-specific LINE-1 element and ZFP69B favoring LTR HERVH-int (Fig. 7D,E). Yet, an examination of their secondary targets identifies L1MC1 at a significant frequency in both cases, suggesting that the ancestral ZFP69 might have recognized this TE. Remarkably HERVH is at most 29 myo, that is, much younger than ZFP69B (Supplemental Fig. S8A). Thus, rather than evolutionary fixation of the KZFP to block a newly emerged ERV, it is the TE that apparently gained from recruiting a pre-existing KZFP. Other examples of KZFPs encoded by neighboring genes and displaying distinct primary but shared secondary targets include *ZNF695*, *ZNF669*, and *ZNF124*, transcribed from a Chr 1 KZFP gene cluster, and *ZNF354A*, *ZNF354B*, *ZNF454*, *ZNF879*, and *ZNF354C*, encoded next to each other on Chr 5 (Supplemental Fig. S8B).

**Figure 8.**
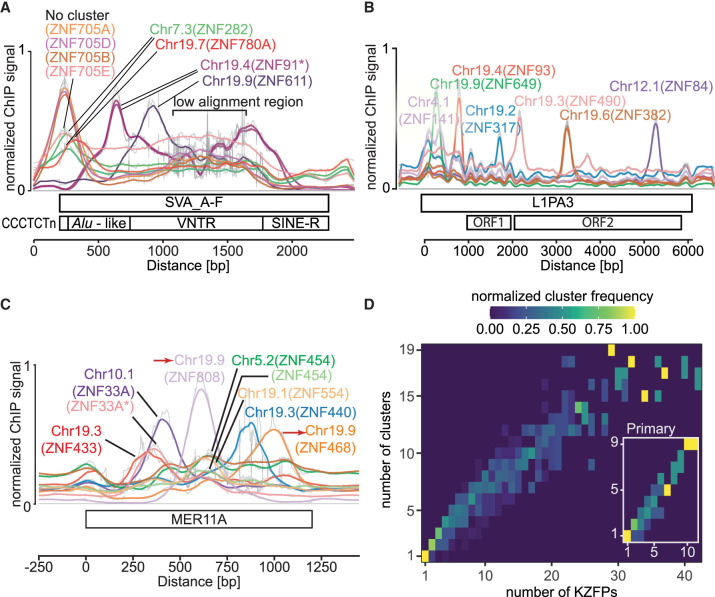
Localization of multiple KZFPs targeting the same TE subfamily. (*A*–*C*) Cluster location of the indicated KZFPs, which primarily bind the respective TE subfamilies. Duplicated clusters are marked with red arrows; external data sets are marked with stars. (*A*) Alignment of approximately 200 of the longest SVA_A to SVA_F elements. (*B*,*C*) Alignment of 1000 L1PA3 and MER11A elements. (*D*) Heatmap comparing the genomic locations of KZFP genes, the products of which target the same TE subfamilies, showing that they are spread across multiple gene clusters. Each square represents the indicated number (*x*-axis) of different KZFPs targeting the same TE subfamily and the number (*y*-axis) of KZFP gene clusters in which these KZFPs are located. Colors represent the frequency with which the number of KZFPs are found in the number of different clusters and are normalized for each column. Main panel: all KZFPs targeting a subfamily (FDR > 0.05). *Inset*: Only KZFPs primarily targeting a subfamily (−log_10_(FDR) within 10% of the highest −log_10_(FDR)).

The recruitment of multiple KZFPs by given TE integrants and strong evidence for the role of differential selective pressures (e.g., the arms race model for ZNF93 and the transition between L1PA3 and L1PA2 vs. the consistent recruitment of ZNF84 and ZNF282 by multiple generations of LINE-1) suggest a multimodal evolution of the TE-KZFP relationship. This is supported by the finding that the same TE subfamily is often recognized by KZFPs encoded in different gene clusters, as exemplified with MER11A, L1PA3, and SVAs (Fig. 8A–C), an observation which can be generalized and leads to an approximately linear relationship between the number of KZFPs involved in recognizing a given element and the number of KZFP gene clusters they are located in, with generally no more than two of these KZFPs being located in the same cluster. This becomes more pronounced when considering only primary targets, where almost every KZFP is located in a separate cluster (Fig. 8D). This dispersed localization of KZFP genes targeting the same TE subfamilies suggests the independent fixation of multiple KZFPs upon emergence of a new TE subfamily, paving the ground for subsequent multimodal evolution of TE-KZFP relationships.

## Discussion

The extensive mapping of human KZFP genomic targets confirms that in their vast majority these proteins recognize sequences embedded in transposable elements. Altogether, genomic binding sites have now been characterized for 358 out of 378 family members, revealing that 254 of them have a TE as their primary target. Conversely, our results indicate that most TEs can be recognized by a KZFP, and many by more than one. Because only ∼1:1000 TEs is still capable of transposition, it lends strong credence to our earlier proposal (Trono 2015) that the evolutionary selection and maintenance of KZFP genes has been geared towards the domestication of TE-embedded regulatory sequences (TEeRS) rather than driven by the need to block the spread of these genetic invaders, even though the two are not mutually exclusive. For instance, ZNF93 is the prototype of a KZFP initially involved in some sort of TE-host arms race, the fixation of which coincided with the emergence of the L1PA6 generation of LINE-1 before their L1PA3 descendants escaped its control by deleting its binding site, some 15 to 20 million years later (Jacobs et al. 2014). Yet, even for ZNF93, control of transposition appears to have been only a temporary function, as this KZFP keeps recruiting a TRIM28-associated repressor complex to the 5′ end of thousands of L1PA6 to L1PA3 integrants, all of which became transposition-defective millions of years ago.

Most remarkable is the ability by many integrants from the LINE, ERV, or SVA families to recruit over different sites in their sequences multiple KZFPs, most bearing a standard KRAB domain with verified repressor potential. Although for ERVs and SVAs these KZFP binding sites are generally clustered close to known promoter or enhancer elements, with for instance a concentration of KZFP-recruiting motifs within ERV LTRs, the distribution of KZFP peaks over the whole sequence of LINE-1 integrants, including in the central and 3′ regions, is intriguing. First, it confirms that transposition-deficient TEs are major genomic docking sites for KZFPs, because many LINE-1 integrants are 5′ deleted, hence devoid of 5′ promoter, because of incomplete reverse transcription. Second, it indicates that these distally situated LINE-1 sequences and their KZFP ligands must accomplish some biological functions, the nature of which is still largely to decipher. Third, it calls for studies examining the spatiotemporal regulation and biological impact of the recruitment of these KZFPs on their TE targets both alone and, when relevant, in combinations. In that respect, it is important to note that, because we aimed at a systematic analysis of the entire human KZFP family performed in one consistent experimental system, we relied exclusively on the overexpression of tagged proteins in 293T cells. Although this has largely turned out to be a valuable first approach to identify the genomic targets of KZFPs on a global level (Jacobs et al. 2014; Najafabadi et al. 2015; Imbeault et al. 2017; Helleboid et al. 2019; Pontis et al. 2019, 2022; Takahashi et al. 2019; Turelli et al. 2020), binding at individual loci is expected to be context dependent and notably influenced by the epigenetic features of specific cells and tissues, warranting targeted analyses. For some KZFPs, we detected only low numbers of ChIP-seq peaks in this system. This could be because of the inaccessibility of some *bona fide* DNA binding sites in HEK293T cells or to KZFPs truly recruited at limited numbers of genomic locations. The detailed binding features of each KZFP tested in our system, given in Supplemental Tables S2 and S3, provide the necessary background to interpret these data and integrate them properly in future analyses.

The evolutionary conservation of individual KZFPs correlates with the genetic constraint imposed on their coding sequences: Older KZFP genes display lower degrees of genetic variation in the human population than their more recent counterparts, notably at positions encoding amino acids predicted to dictate the DNA binding specificity of their products. Yet the target sequences of these highly conserved KZFPs reveal an interesting dichotomy, encompassing both very old TEs such as L2 or DNA transposons and evolutionary recent elements such as SVAs or ERV1s. Furthermore, many of these KZFPs harbor variant KRAB domains that do not interact with TRIM28 and associated epigenetic modifiers but with other types of protein complexes, and are devoid of repressor activity (Helleboid et al. 2019; Tycko et al. 2020). This strongly supports a model whereby TEs serve as vectors of *cis*-acting regulatory sequences of a broad functional diversity.

In contrast to older family members, evolutionarily recent KZFPs almost universally target TEs, often have paralogs, and display a TRIM28-centered protein interactome primarily consistent with transcriptional repression (Helleboid et al. 2019; Tycko et al. 2020). The greater degree of polymorphism observed in the human population at positions determining the genomic targets of these recently emerged KZFPs may be explained by the absence of TEs forcing fixation of at least part of their ZF-coding sequences, or to at least partial redundancy in the action of paralogs. Differentials in coding constraint varied within unequivocally identified sets of KZFP paralogs, being very narrow in some cases (e.g., *ZNF75A* and *ZNF75D*; *ZFP30* and *ZFP82*) and quite broad in others (e.g., *ZNF160* and *ZNF665*; *ZNF181* and *ZNF302*). No single parameter could account for these differences. For instance, ZNF75A and ZNF75D both recognize the 3′ end of KZFP genes, whereas ZFP30 and ZFP82, respectively, bind LINEs and SINEs, that is, completely distinct sets of genomic targets. As well, ZNF679-ZNF716 and ZNF600-ZNF611 are two pairs of evolutionarily recent (<20 myo) paralogs, yet they present with coding constraint differentials that are negligible for the former and pronounced for the latter. Still, it is noteworthy that for paralogs of detectably distinct ages, the older gene is generally more constrained than its duplication product, recapitulating a trend noted for the entire KZFP family.

The KZFP gene pool of a lineage undergoes a high evolutionary turnover, as indicated by the mammalian and primate specificity of 88% and 32%, respectively, of human family members. The gene duplication mechanism underlying this phenomenon allows for an efficient diversification of the *trans*-regulatory space without losing track of physiology, as new TEeRS emerging by genetic drift of the host TE pool can be controlled and potentially exploited without unleashing the perturbation potential of older TEeRS. This smooth transition model is supported by an examination of the secondary targets of paralogs such as ZFP69 and ZFP69B, which suggests that an ancestral *ZFP69* gene targeting L1MC elements duplicated to have the original gene conserve its affinity for this TE and the zinc fingerprint of its copy drift to become fixed upon recognition of a later emerged HERV. In this system, even if only a fraction of newcomer genes ends up positively selected, a rapid flux of new candidates, on both the TE and KZFP sides, fuels the evolution of a lineage's regulome. Most frequently, because of environmental and physiological constraints, this will result in purely mechanistic speciation, with conservation of biological processes but turnover of some of their *cis*- (the TEeRS) and *trans*- (the KZFPs) regulators, as during early embryogenesis or gametogenesis (Pontis et al. 2019, 2022; Barnada et al. 2022; Xiang et al. 2022). Occasionally, however, it may give rise to new traits, notably in organ systems where the range of phenotypes compatible with reproductive life hence *trans*-generational inheritance is greater, as suggested by the increasingly recognized importance of TE/KZFP-mediated regulation in the developing human brain (Nowick et al. 2009; Farmiloe et al. 2020; Turelli et al. 2020; Playfoot et al. 2021, 2022; Johansson et al. 2022; Patoori et al. 2022).

## Methods

### Census of the human KRAB-zinc finger protein clusters

KZFP pairs were detected and their age defined as described in Imbeault et al. (2017). In short, the human genome (hg19) was translated in six reading frames and scanned for zinc finger and KRAB domains using Hidden-Markov-Models (Pfam [El-Gebali et al. 2019]: KRAB [PF01352] and zf-C2H2 [PF00096]). Hits for KRAB and zinc finger domains were combined based on proximity and strandness and then manually curated and integrated with existing gene or pseudogene annotations. KZFP ages were determined as described in Imbeault et al. (2017), comparing their DNA-interacting amino acids (zinc fingerprints) across and between species (see Supplemental Fig. S8A; Supplemental Methods for further information). Paralog definitions were obtained from Imbeault et al. (2017) and are based on clustering using a 60% identity between zinc fingerprints as a cutoff. The KZFP clusters were defined as having at least three KZFPs that are no more than 250 kb apart from the center of another member, consistent with Huntley et al. (2006). The clusters are named after their chromosome and then numbered starting from the short arm of the chromosome. The size of chromosomes and positions of centromeres were taken from UCSC Genome Browser annotation data for hg19 (Haeussler et al. 2019).

### Primate phylogeny and natural selection

The time of divergence (i.e., branch lengths) between human, chimpanzee, gorilla, orangutan, macaque, marmoset, tarsier, galago (a.k.a. bush baby), and mouse lemur was obtained from 10kTrees, which uses Bayesian inference to estimate these (Arnold et al. 2010). Measures of natural selection in terms of *d*_N_/*d*_S_ across the nine primate species listed above was obtained with PAML (v4.4) as previously described (McLaren et al. 2015).

### Human genetic variation data

Human genetic exome and whole genome sequencing data were obtained from The Genome Aggregation Database (gnomAD) (Lek et al. 2016; Karczewski et al. 2020) (release-2.0.2) for 123,136 and 15,496 individuals, respectively. The released genetic data was processed and filtered through several steps to guarantee that only high-quality variants were included. First, all variants ±1 kb around the KZFP canonical transcripts, as defined by Ensembl (v75, hg19) (Cunningham et al. 2022), were extracted and filtered for variant quality, thus only retaining variants annotated as “PASS”. Second, all indels were normalized and multiallelic variants split using BCFtools (v1.8) (Danecek et al. 2021) and reannotated with the Variant Effect Predictor (McLaren et al. 2016) and LOFTEE (v0.3beta) (Karczewski et al. 2020). Third, all missense and LoF variants, defined as either frameshift, stop-gain, or splice variants, were extracted from both the exome and whole genome data sets and either low confidence or flagged LoF variants were removed. The latter was primarily because of LoF variants found in the last 5% of the canonical transcript. Because genomic sequencing methods can yield variable coverage of genetic regions, especially when it comes to exome sequencing that is dependent on the capture of previously annotated protein-coding genes, we excluded all canonical transcripts having an average per-base coverage < 20×. Thus, bringing the total number of included KZFPs to 361. Furthermore, exons with an average per-base coverage < 20× were also removed, and the lengths of the coding sequences used later for normalizations were adjusted accordingly. Finally, the filtered exome and genome data sets were combined, and the allele counts and frequencies for all variants were recalculated, before the removal of all singletons (allele count = 1) to hinder inflation of observed mutational events because of potential technical artifacts.

### Domain and site specifications

The genomic positions of the C2H2 zinc finger domains were obtained from the Ensembl database (v75, hg19) (Cunningham et al. 2022). For each KZFP, only the ones from the canonical transcripts (as defined by Ensembl) were considered. The positions of the specific amino acids within these domains were computationally annotated. *Z* scores for the cysteine and histidine (C2H2) residues were calculated with the number of missense variants normalized to the number of zinc finger domains within the canonical transcript of each KZFP. For missense and LoF variants spanning either the whole CDS or a full protein domain, the number of variants per gene, x, was normalized by the length of the canonical coding sequence before *Z*-score transformations.

$$Z\;score=\frac{\left(x-mean(variantcount)\right)}{sd(variantcount)}.$$

### Cell lines

HEK293T cells overexpressing HA-tagged KZFPs were generated as described in Imbeault et al. (2017). In short, cDNAs from the human KZFPs were codon-optimized and synthesized using the GeneArt service from Thermo Fisher Scientific (former Life Technologies). Sequences were cloned into the doxycycline inducible expression vector pTRE-3HA which yields C-terminally tagged proteins. Stable cell lines were generated using Lentivector transduction of mycoplasma free HEK293T cells as described on http://tronolab.epfl.ch. Presence and integrity of the integrated plasmids were verified using Sanger sequencing (primers: CMV1f: GGAGGCCTATATAAGCAGAGCTCGT, PGK4b: CGAACGGACGTGAAGAATGTGCGAGA) and KZFP expression was verified via Western blot with an anti-HA antibody (ref. 12013819001, Roche). HEK293T cells were chosen in order to have a consistent cell line and genomic background for all conducted experiments.

### ChIP-seq

Chromatin was prepared as described in Imbeault et al. (2017) and ChIP-seq was performed as described in Iouranova et al. (2022). In short: 30 million KZFP expressing HEK293T cells were used after induction for more than 48 h with 1 ng/mL doxycycline. Cells were cross-linked with 1% methanol free formaldehyde for 10 min before nuclear extraction followed by sonication in a Covaris E220 sonicator resulting in DNA fragments between 200–500 bp. IP was performed overnight using 15 µg anti-HA.11 antibody (BioLegend ref: 901503) coupled to 75 uL Dynabeads Protein G (Invitrogen ref: 10009D). 10 ng of material for both total inputs and chromatin immunoprecipitated samples was used for library preparation. After end-repair and A-tailing, Illumina IDT indexes were ligated to the samples. Aliquots were tested in qPCR to determine the optimal number of PCR cycles needed to amplify each library without reaching saturation. Libraries were size-selected using Ampure XP beads (Beckman Coulter), quality-checked on a Bioanalyzer DNA high sensitivity chip (Agilent), and quantified with a Qubit dsDNA HS assay kit (Qubit 2.0 Fluorometer, Invitrogen) using Illumina adapters. Libraries were sequenced as indicated at the NCBI Gene Expression Omnibus (GEO: https://www.ncbi.nlm.nih.gov/geo/) under accession number GSE200964 producing either 100 bp single-end or 75 bp paired-end reads.

### Processing of ChIP-seq and ChIP-exo data

Both previously published (Imbeault et al. 2017; Helleboid et al. 2019) and new data were processed together. Reads were mapped to the human genome assembly hg19 using Bowtie 2 short read aligner v2.3.5.1 (Langmead and Salzberg 2012), using the ‐‐sensitive-local parameter. Prior to peak calling the following reads were removed: multimapped reads (MAPQ < 10), blacklisted regions, and regions with high levels in input samples (Grey List) defined by the R (R Core Team 2022) package GreyListChIP (https://bioconductor.org/packages/GreyListChIP/). Peaks were called using MACS2 v2.2.4 (Zhang et al. 2008) with default parameters except for *-q 0.01* and ‐‐*keep-dup all*. For ChIP-seq experiments a batch specific total input file was used; for ChIP-exo data the approach described in Imbeault et al. (2017) of random sampling all experiments was used to generate a total input. Performing the analyses including multimapped reads slightly changed the set of peaks, adding some and removing others, but did not influence the vast majority of target enrichments (Supplemental Fig. S9A–D). Motives were taken from Cis-BP (Weirauch et al. 2014) whenever possible or identified using RSAT and the peak-motifs function (Thomas-Chollier et al. 2012).

### External ChIP-seq data

To find KZFP ChIP-seqs performed by others, the programmatic access to GEO eSearch and eFetch functions were used (Barrett et al. 2013) to search and retrieve submissions containing any KZFP name but not the keywords “RNA” or “H3K”. The resulting hits were then manually curated using the GEOquery (https://bioconductor.org/packages/GEOquery/) R package (R Core Team 2022) in order to get BED files from ChIP-seq experiments. Peaks not called on hg19 were lifted over to hg19 using liftOver from rtracklayer (https://bioconductor.org/packages/rtracklayer/) and chain files from UCSC (Haeussler et al. 2019).

### Enrichment on repeats

Repeat enrichment analyses from ChIP data were performed using pyTEnrich (https://alexdray86.github.io/pyTEnrich). Repeat annotations were obtained from UCSC (http://hgdownload.soe.ucsc.edu/goldenPath/hg19/database/rmsk.txt.gz). As the entire genome was not mappable in the experiments, the size of the mappable genome specific to these ChIP experiments needed to be calculated to accurately estimate the null hypothesis of how many times a peak would overlap a repeat by chance. To do so, regions with zero coverage were identified for all ChIP-seq and ChIP-exo using BEDTools genomcov (Quinlan and Hall 2010), merging regions with <100 bp distance to reduce computational load. The resulting files were combined using BEDTools intersect (Quinlan and Hall 2010) to generate a single set of regions which have zero coverage across all experiments. These regions were then filtered to be bigger than 40 kb to reduce computational load as the influence of smaller regions on enrichments were negligible. The resulting zero coverage regions as well as the Y Chromosome, which is absent in HEK293T cells, were removed from the reference genome by pyTEnrich to generate more accurate statistics for the enrichments. Enrichments with FDR < 0.05 are considered significant. To normalize FDR between experiments −log_10_(FDR) were divided by their maximum yielding a scale from 0 to 1 or least to most enriched, values above 0.9 on this scale are considered primary targets.

### Multiple sequence alignment and line plots

MSA plots were made as described in Iouranova et al. (2022). In short: FASTA sequences for the indicated subfamilies were extracted from the hg19 genome assembly, aligned individually using MAFFT (Katoh and Standley 2013) with parameters ‐‐reorder ‐‐auto, and then merged together using MAFFT's -merge option. To increase readability, positions in the alignment (columns) with more than 85% gaps were removed. To capture signal at the border the alignments are extended by 200–500 bp of unaligned sequences. ChIP-seq and -exo signals are scaled for each line (row) to the [0,1] interval before being superimposed on the alignments. Average ChIP-seq signals across all rows are plotted on top of the alignments or without alignment for Figures 6 and 8. Motifs were taken from Cis-BP (Weirauch et al. 2014), converted to position weight matrixes, and scanned for in the human genome (hg19) using PWMscan (Ambrosini et al. 2018) with default settings. Line plots in Figure 3B were generated using deepTools plotProfile (Ramírez et al. 2016). SVAs for all subfamilies (A–F) were centered on a well-conserved region on the edge of the VNTR with the consensus sequence ACTAAGAAAAATTCTTCTGCCTTGGG.

### Reference genome

Analyses shown throughout this publication were performed using the reference genome version hg19 for the sake of consistency. Analyses of the ChIP-seq data were performed in hg19 and GRCh38 in parallel with no major impact on the results. To facilitate future analyses, peak files for all experiments are available for download on the KRABopedia database in both hg19 and GRCh38.

## Data access

All raw and processed sequencing data generated in this study have been submitted to the NCBI Gene Expression Omnibus (GEO; https://www.ncbi.nlm.nih.gov/geo/) under accession number GSE200964. Cumulated information on each human KZFP is available at the KRABopedia (https://tronoapps.epfl.ch/web/krabopedia/).

## Supplementary Material

Supplement 1

Supplement 2

Supplement 3

Supplement 4

Supplement 5
