# Supplementary material for: Genetic features and genomic targets of human KRAB-zinc finger proteins
Source: Genome Res. 2023 Aug;33(8):1409–23. doi: 10.1101/gr.277722.123 (PMC10547255; doi:10.1101/gr.277722.123)
Supplement: Supplement 5 [file Supplemental_Material.pdf]

Contents

Supplementary Figures

Figure S1: Age and polymorphism of KZFPs in clusters

Figure S2: Variable constraint within each KZFP cluster

Figure S3: Comparison of ZNF160 and ZNF665 paralogs

Figure S4: Replicate comparison

Figure S5: Targets of human KZFPs, external data

Figure S6: Example of Primary vs. Secondary targets

Figure S7: ZNF141 is binding to SVA VNTR

Figure S8: Evolutionary history of paralogs and additional examples of shared targets

Figure S9: Impact of multi-mapped reads on peak calling and target enrichment

Supplementary Methods

Cross-species zinc fingerprint comparison

Supplementary Tables (S1-S4: .xlsx files)

Table S1: Census of human KZFPs

Table S2: ChIP-seq data on human KZFPs

Table S3: Primary targets of KZFPs

Table S4: KZFP and TE age comparison

Supplementary References

Supplementary Figures

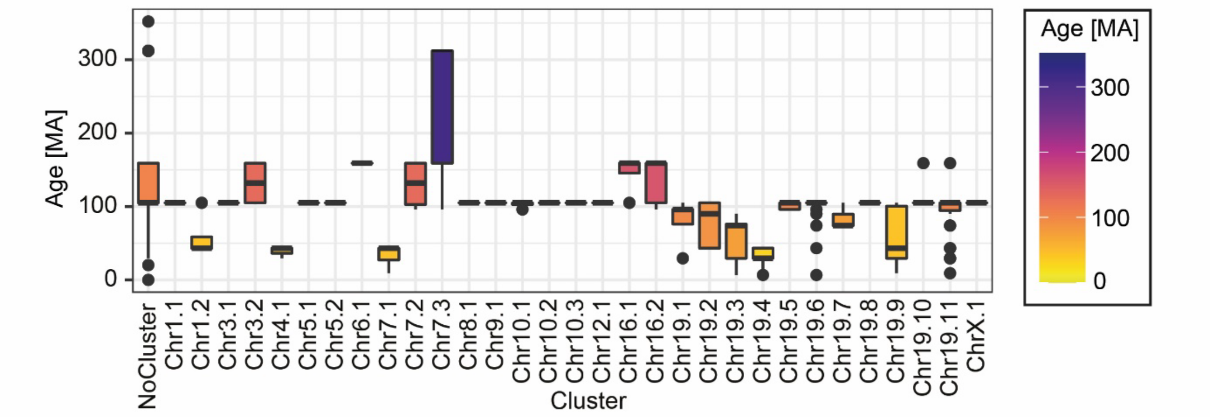

Figure S1: Age and polymorphism of KZFPs in clusters

Boxplots of the ages per cluster as defined in Figure 1, colored by the median age of the KZFPs in the clusters.

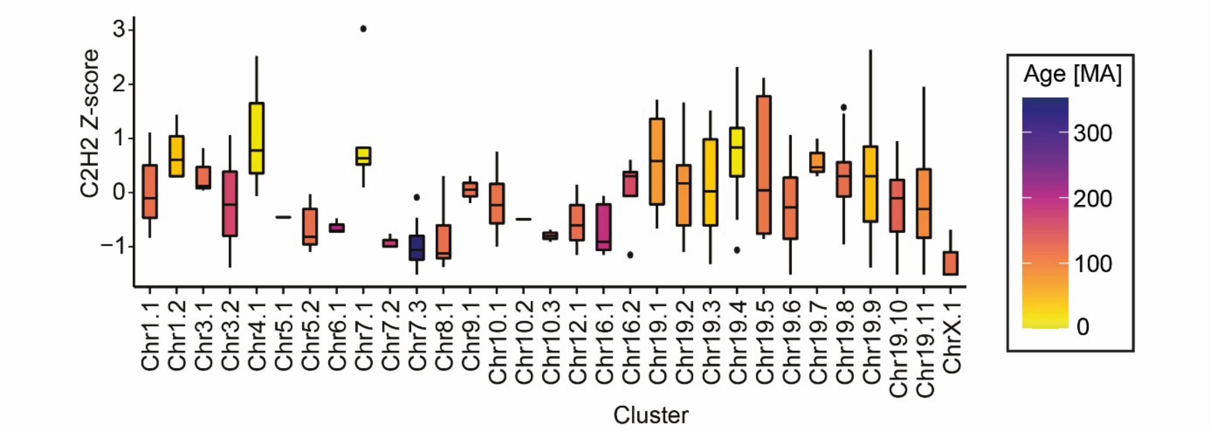

Figure S2: Variable constraint within each KZFP cluster

Boxplots of the variable levels of constraint in the cysteine and histidine (C2H2) levels across KZFP clusters, colored by the mean age of the KZFPs within each genomic cluster.

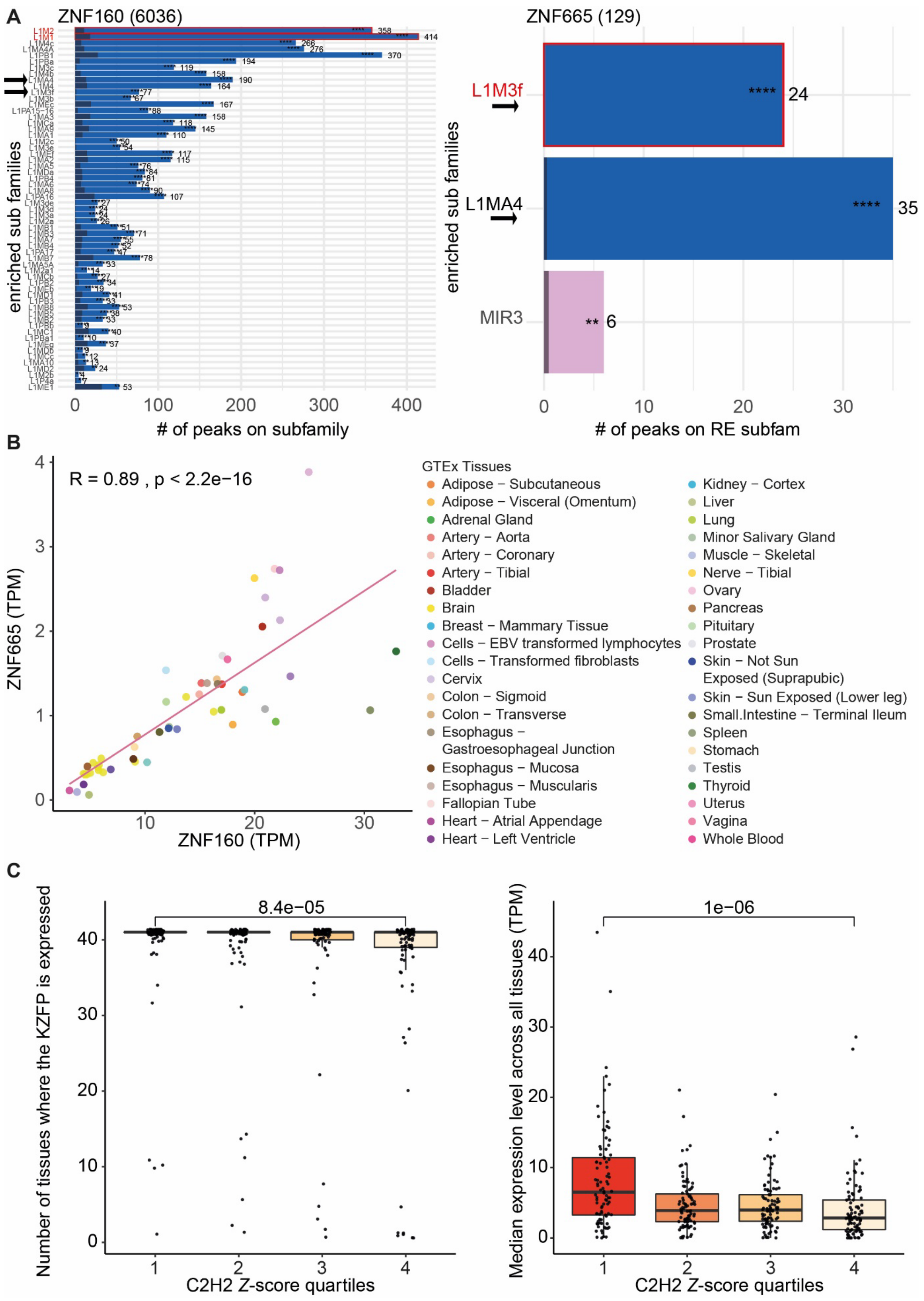
A
ZNF160 (6036)
enriched sub families
# of peaks on subfamily
ZNF665 (129)
L1M3f
L1MA4
MIR3
enriched sub families
# of peaks on RE subfam
B
R = 0.89 , p < 2.2e−16
ZNF665 (TPM)
ZNF160 (TPM)
GTEx Tissues
Adipose − Subcutaneous
Adipose − Visceral (Omentum)
Adrenal Gland
Artery − Aorta
Artery − Coronary
Artery − Tibial
Bladder
Brain
Breast − Mammary Tissue
Cells − EBV transformed lymphocytes
Cells − Transformed fibroblasts
Cervix
Colon − Sigmoid
Colon − Transverse
Esophagus − Gastroesophageal Junction
Esophagus − Mucosa
Esophagus − Muscularis
Fallopian Tube
Heart − Atrial Appendage
Heart − Left Ventricle
Kidney − Cortex
Liver
Lung
Minor Salivary Gland
Muscle − Skeletal
Nerve − Tibial
Ovary
Pancreas
Pituitary
Prostate
Skin − Not Sun Exposed (Suprapubic)
Skin − Sun Exposed (Lower leg)
Small.Intestine − Terminal Ileum
Spleen
Stomach
Testis
Thyroid
Uterus
Vagina
Whole Blood
C
8.4e−05
Number of tissues where the KZFP is expressed
C2H2 Z-score quartiles
1e−06
Median expression level across all tissues (TPM)
C2H2 Z-score quartiles

Figure S3: Comparison of ZNF160 and ZNF665 paralogs

A) Similar genomic targets for ZNF160 and ZNF665. Enrichments of ZNF160 and ZNF665 peaks over different repetitive element subfamilies (FDR < 0.01). The width of the colored bars represents the number of peaks per subfamily also shown as a number on the right of the bar. The black transparent bars represent the expected number of peaks following a random distribution. The FDR of the enrichment is shown with stars (FDR < 0.0001 = ****, < 0.001=***, < 0.01=**, < 0.05=*, >= 0.05 = n.s). The y-axis is ordered by FDR. The number next to the title indicates the total number of peaks for the experiment. Primary targets for each KZFP are highlighted in red. (B) Expression levels in transcripts per million (TPM) of ZNF160 and ZNF665 across all tissues depicted in GTEx (GTEx Consortium 2017). C) Biological consequences of KZFP constraint. Left: KZFPs in the highest quartile of C2H2 constraint are expressed in more tissues than KZFPs in the lowest quartile. Right: The mean expression levels of KZFPs in the first quartile of C2H2 constraint is higher than for those KZFPs in the fourth constraint quartile (lowest constraint). All p-values are from Wilcoxon rank sum tests.

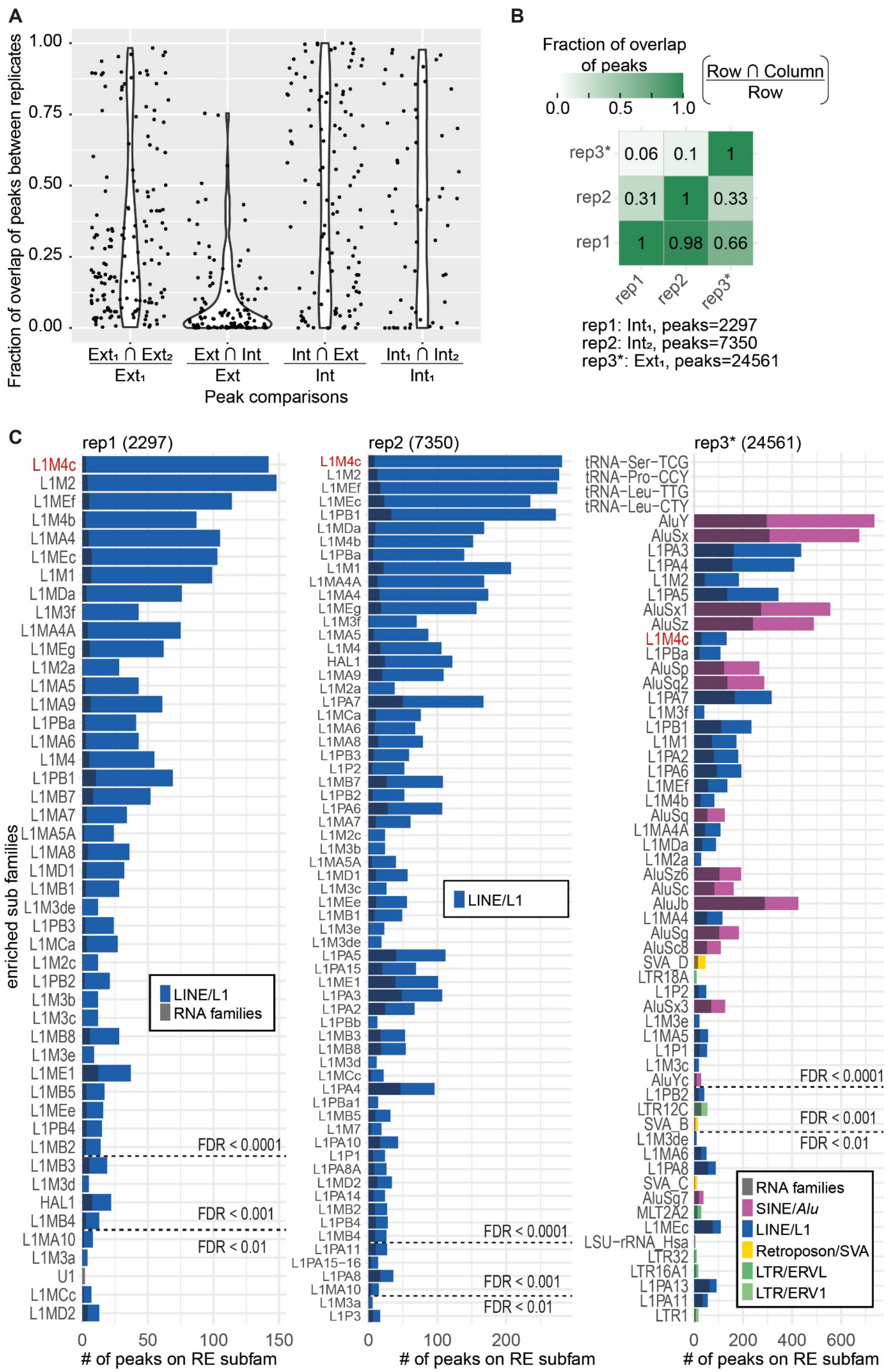
A
Fraction of overlap of peaks between replicates
1.00
0.75
0.50
0.25
0.00
Ext₁ ∩ Ext₂ / Ext₁
Ext ∩ Int / Ext
Int ∩ Ext / Int
Int₁ ∩ Int₂ / Int₁
Peak comparisons
B
Fraction of overlap of peaks
0.0 0.5 1.0
Row ∩ Column / Row
rep3* 0.06 0.1 1
rep2 0.31 1 0.33
rep1 1 0.98 0.66
rep1 rep2 rep3*
rep1: Int₁, peaks=2297
rep2: Int₂, peaks=7350
rep3*: Ext₁, peaks=24561
C
rep1 (2297)
rep2 (7350)
rep3* (24561)
enriched sub families
LINE/L1
RNA families
SINE/Alu
Retroposon/SVA
LTR/ERVL
LTR/ERV1
FDR < 0.0001
FDR < 0.001
FDR < 0.01
# of peaks on RE subfam

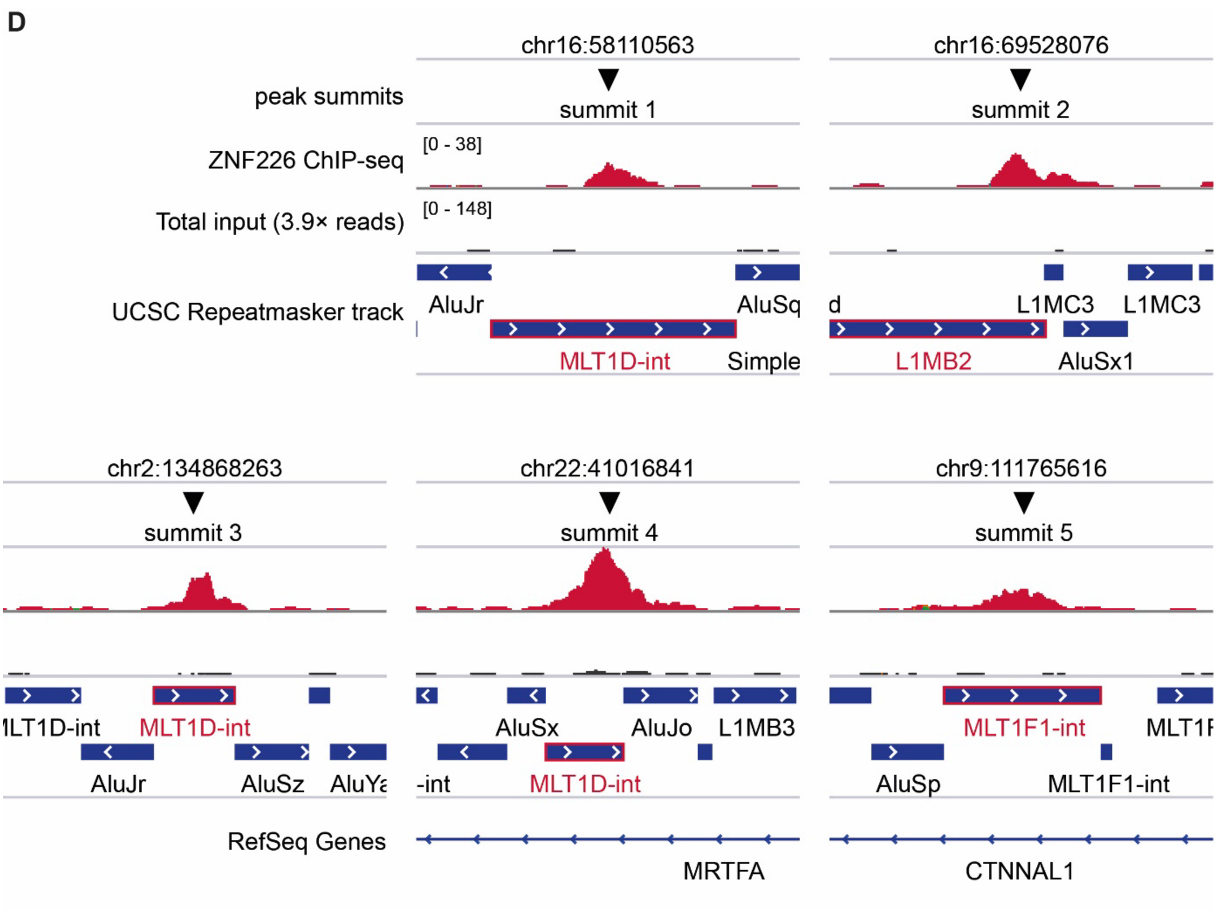

Figure S4: Replicate comparison

A) Violine plots with comparisons of the fraction of overlap of all ChIP-seq and ChIP-exo experiments with replicates; between internal (Int) experiments performed and analyzed as described in the Methods section and external (Ext) datasets with heterogenous experimental and analysis procedures. Each dot represents a comparison of the peaks in two experiments performed on the same KZFP. The different comparisons from left to right are: 1) Comparisons between external datasets, 2) comparisons between external and internal datasets (i.e., what fraction of peaks from external dataset can be found in internal replicates), 3) comparisons between internal and external datasets (i.e., what fraction of peaks from internal dataset can be found in external replicates) and 4) comparisons between internal replicates. B) Example of peak comparisons across replicates for ZNF136. A matrix showing the fraction of overlap of peaks between different experiments for ZNF136 on the rows with the columns. Experiments are indicated below as either being internal (Int) or external (Ext) as well as the number of

peaks per experiment. Rep1 is a new dataset generated in for this paper, rep2 was previously generated together with the data published in (Imbeault et al. 2017) but not published at the time, rep3 is an external dataset (marked with a star) generated and analyzed by (Schmitges et al. 2016). C) Enrichments of ZNF136 rep1, rep2 and rep3 over different repetitive element subfamilies (FDR < 0.01). The positions of L1M4c are highlighted in red. The width of the colored bars represents the number of peaks per subfamily. The black transparent bars represent the expected number of peaks following a random distribution. The areas on the plots with different FDRs of the enrichment are delineated with dashed lines. The y-axis is ordered by FDR. The number next to the title indicates the total number of peaks for the experiment. D) Genome browser tracks from all five peaks for ZNF226. Shown from top to bottom are: the summits of the peaks called peaks, the ChIP-seq signal, the total input signal (with the data range increased 3.9× as the input has 3.9× more reads than ZNF226 with 55 mio. compared to 14 mio. reads), the UCSC Repeatmasker track with the TE overlapping the summit highlighted in red and the RefSeq Genetrack.

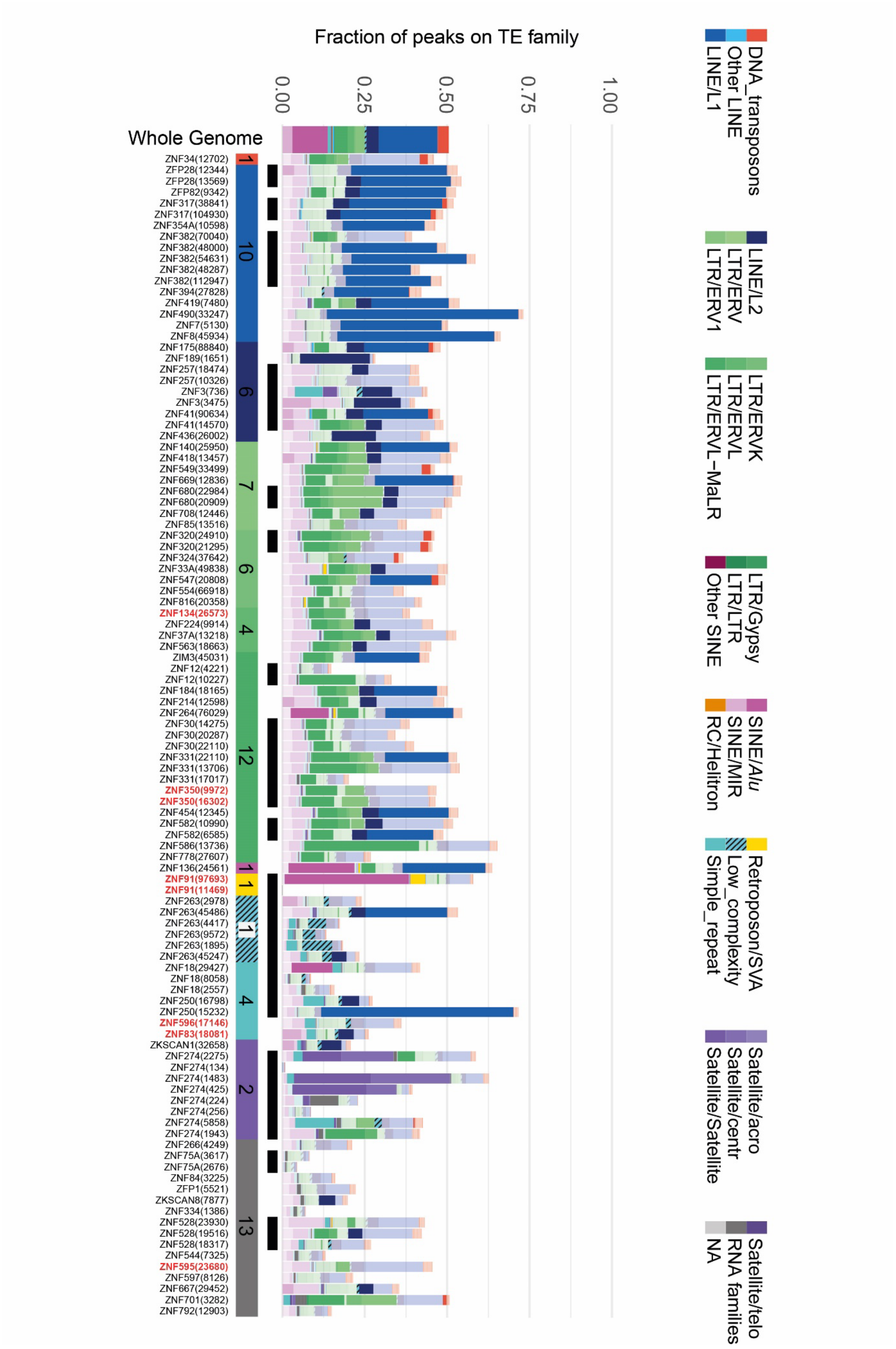
Fraction of peaks on TE family
0.00
0.25
0.50
0.75
1.00
Whole Genome
ZNF34(12702)
ZFP28(12344)
ZFP28(13569)
ZFP82(9342)
ZNF317(38841)
ZNF317(104930)
ZNF354A(10598)
ZNF382(70040)
ZNF382(48000)
ZNF382(54631)
ZNF382(48287)
ZNF382(112947)
ZNF394(27828)
ZNF419(7480)
ZNF490(33247)
ZNF7(5130)
ZNF8(45934)
ZNF175(88840)
ZNF189(1651)
ZNF257(18474)
ZNF257(10326)
ZNF3(736)
ZNF3(3475)
ZNF41(90634)
ZNF41(14570)
ZNF436(26002)
ZNF140(25950)
ZNF418(13457)
ZNF549(33499)
ZNF669(12836)
ZNF680(22984)
ZNF680(20909)
ZNF708(12446)
ZNF85(13516)
ZNF320(24910)
ZNF320(21295)
ZNF324(37642)
ZNF33A(49838)
ZNF547(20808)
ZNF554(66918)
ZNF816(20358)
ZNF134(26573)
ZNF224(9914)
ZNF37A(13218)
ZNF563(18663)
ZIM3(45031)
ZNF12(4221)
ZNF12(10227)
ZNF184(18165)
ZNF214(12598)
ZNF264(76029)
ZNF30(14275)
ZNF30(20287)
ZNF30(22110)
ZNF331(22110)
ZNF331(13706)
ZNF331(17017)
ZNF350(9972)
ZNF350(16302)
ZNF454(12345)
ZNF582(10990)
ZNF582(6585)
ZNF586(13736)
ZNF778(27607)
ZNF136(24561)
ZNF91(97693)
ZNF91(11469)
ZNF263(2978)
ZNF263(45486)
ZNF263(4417)
ZNF263(9572)
ZNF263(1895)
ZNF263(45247)
ZNF18(29427)
ZNF18(8058)
ZNF18(2557)
ZNF250(16798)
ZNF250(15232)
ZNF596(17146)
ZNF83(18081)
ZKSCAN1(32658)
ZNF274(2275)
ZNF274(134)
ZNF274(1483)
ZNF274(425)
ZNF274(224)
ZNF274(256)
ZNF274(5858)
ZNF274(1943)
ZNF266(4249)
ZNF75A(3617)
ZNF75A(2676)
ZNF84(3225)
ZFP1(5521)
ZKSCAN8(7877)
ZNF334(1386)
ZNF528(23930)
ZNF528(19516)
ZNF528(18317)
ZNF544(7325)
ZNF595(23680)
ZNF597(8126)
ZNF667(29452)
ZNF701(3282)
ZNF792(12903)
1
10
6
7
6
4
12
1
1
1
4
2
13
DNA_transposons
Other LINE
LINE/L1
LINE/L2
LTR/ERV
LTR/ERV1
LTR/ERVK
LTR/ERVL
LTR/ERVL-MaLR
LTR/Gypsy
LTR/LTR
Other SINE
SINE/Alu
SINE/MIR
RC/Helitron
Retroposon/SVA
Low_complexity
Simple_repeat
Satellite/acro
Satellite/centr
Satellite/Satellite
Satellite/telo
RNA families
NA

Figure S5: Targets of human KZFPs, external data

Bar graph showing the fraction of peaks over repetitive element (RE) families for experiments conducted using different over-expression protocols (Table S2). Columns are ordered by the most enriched family which are indicated by the horizontal bar below, along with the number of KZFPs for each category. Replicate experiments are indicated by black squares above the horizontal bar. Significant enrichments (FDR < 0.05) are show in fully opaque colors where non-significant enrichments are transparent. The leftmost bar shows the genome occupancy of all RE families. The total number of peaks per experiment is indicated in brackets after the KZFP name below each bar. KZFPs that are not represented in Figure 5A are highlighted in red.

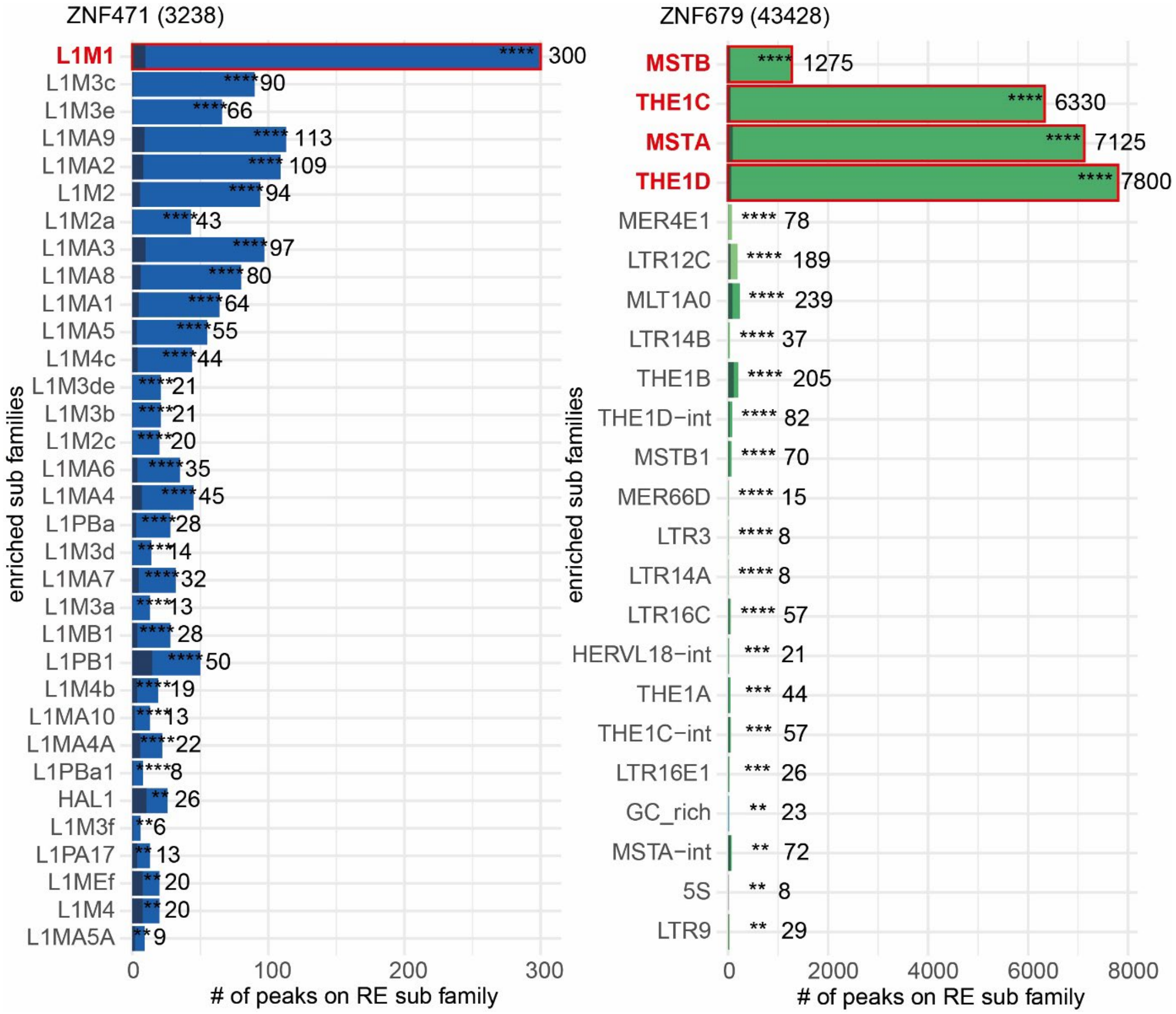

Figure S6: Example of Primary vs. Secondary targets

Enrichments of ZNF471 and ZNF679 peaks over different repetitive element subfamilies (FDR < 0.01). Primary targets for each KZFP are highlighted in red. The width of the colored bars represents the number of peaks per subfamily also shown as a number on the right of the bar. The black transparent bars represent the expected number of peaks following a random distribution. The FDR of the enrichment is shown with stars (FDR < 0.0001 = ****, < 0.001=***, < 0.01=**, < 0.05=*, >= 0.05 = n.s). The y-axis is ordered by FDR. The number next to the title indicates the total number of peaks for the experiment.

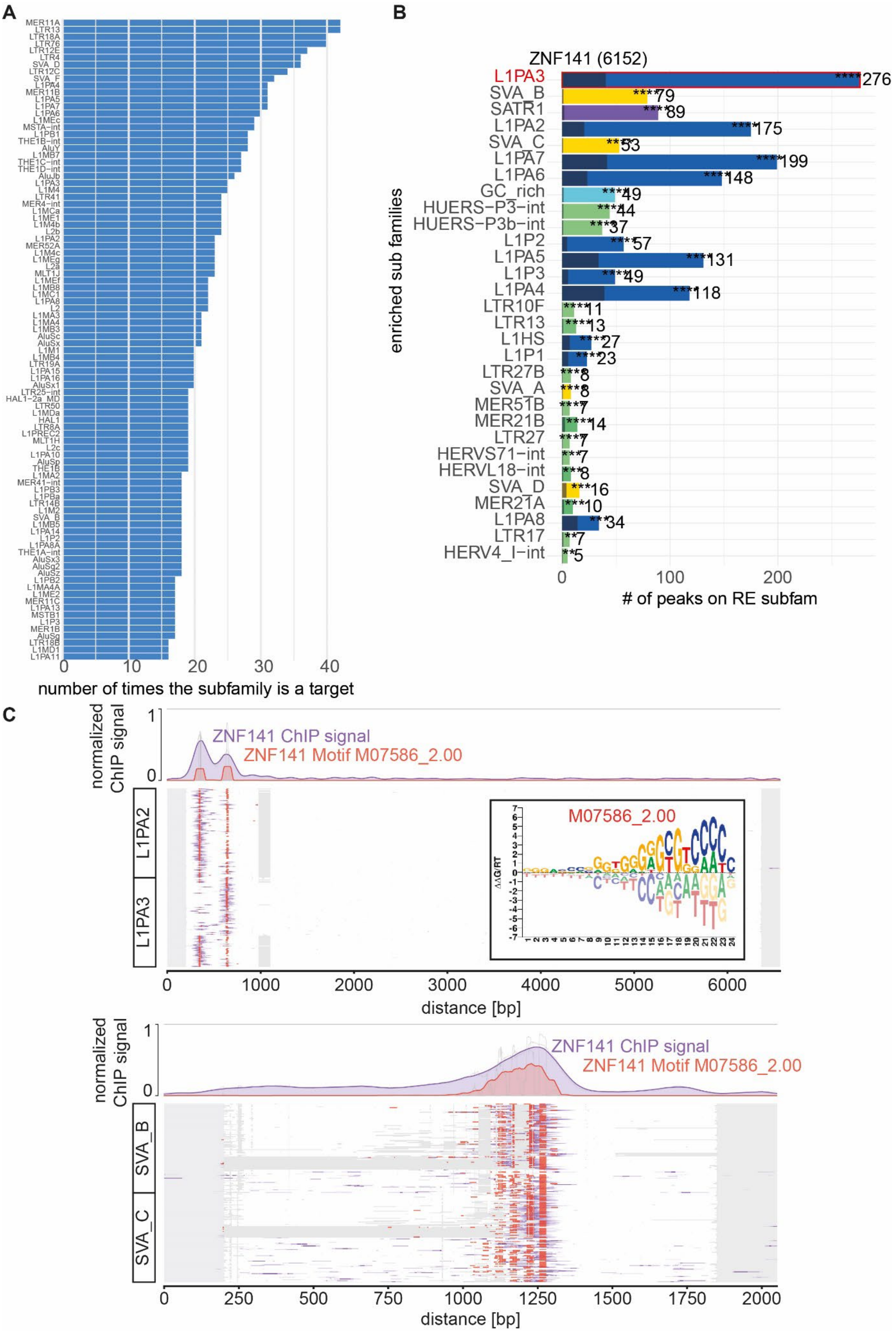
A
number of times the subfamily is a target
0
10
20
30
40
B
ZNF141 (6152)
enriched sub families
L1PA3
SVA_B
SATR1
L1PA2
SVA_C
L1PA7
L1PA6
GC_rich
HUERS-P3-int
HUERS-P3b-int
L1P2
L1PA5
L1P3
L1PA4
LTR10F
LTR13
L1HS
L1P1
LTR27B
SVA_A
MER51B
MER21B
LTR27
HERVS71-int
HERVL18-int
SVA_D
MER21A
L1PA8
LTR17
HERV4_I-int
# of peaks on RE subfam
0
100
200
C
normalized ChIP signal
ZNF141 ChIP signal
ZNF141 Motif M07586_2.00
L1PA2
L1PA3
M07586_2.00
distance [bp]
SVA_B
SVA_C
distance [bp]

Figure S7: ZNF141 is binding to SVA VNTR

A) Bar graph with the number of KZFPs enriched on TE subfamilies (rows). TE subfamilies with more than 15 enriched KZFPs with an FDR < 0.05 are shown. B) Enrichment of ZNF141 peaks over different repetitive element subfamilies (FDR < 0.01). The width of the colored bars represents the number of peaks per subfamily also shown as a number on the right of the bar. The black transparent bars represent the expected number of peaks following a random distribution. The FDR of the enrichment is shown with stars (FDR < 0.0001 = ****, < 0.001=***, < 0.01=**, < 0.05=*, >= 0.05 = n.s). The y-axis is ordered by FDR. The number next to the title indicates the total number of peaks for the experiment. C) Multiple sequence alignment (MSA) over the most enriched targets L1PA2 and 3 (top) and SVA_B and C in (bottom). Up to 200 elements for the indicated targets where aligned, selecting first elements overlapping with a peak and then the longest elements. The signal of the ZNF141 ChIP was laid over the alignment in purple. The locations of the motif identified in (Weirauch et al. 2014) is shown in red. The motif is shown on the right of the top panel. The average signal normalized for each element (row-wise) can be seen as a line plot above the MSA plots.

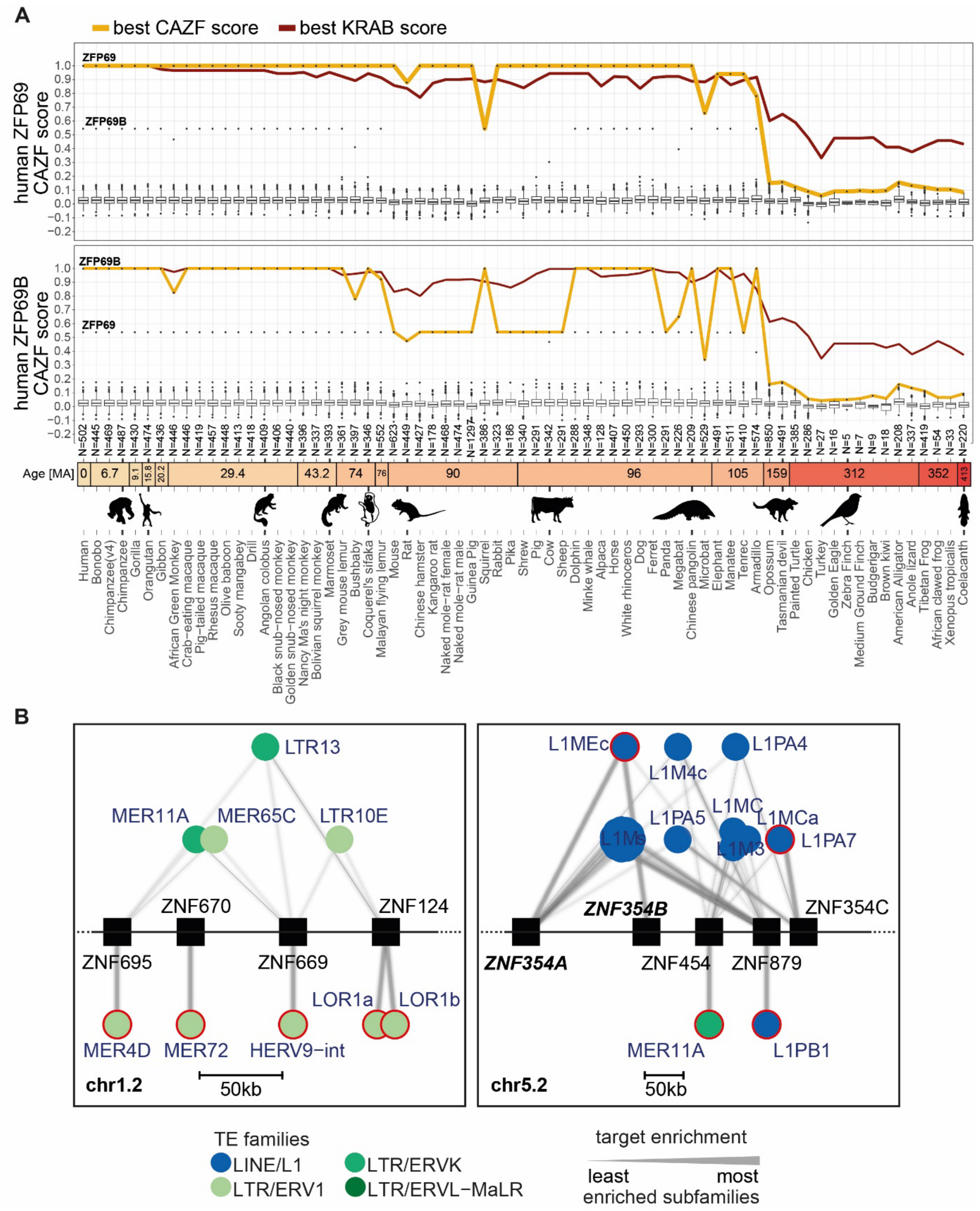
A
best CAZF score
best KRAB score
human ZFP69 CAZF score
ZFP69
ZFP69B
human ZFP69B CAZF score
ZFP69B
ZFP69
Age [MA]
0
6.7
9.1
15.8
20.2
29.4
43.2
74
76
90
96
105
159
312
352
413
Human
Bonobo
Chimpanzee(v4)
Chimpanzee
Gorilla
Orangutan
Gibbon
African Green Monkey
Crab-eating macaque
Pig-tailed macaque
Rhesus macaque
Olive baboon
Sooty mangabey
Drill
Angolan colobus
Black snub-nosed monkey
Golden snub-nosed monkey
Nancy Ma's night monkey
Bolivian squirrel monkey
Marmoset
Grey mouse lemur
Bushbaby
Coquerel's sifaka
Malayan flying lemur
Mouse
Rat
Chinese hamster
Kangaroo rat
Naked mole-rat female
Naked mole-rat male
Guinea Pig
Squirrel
Rabbit
Pika
Shrew
Pig
Cow
Sheep
Dolphin
Minke whale
Alpaca
Horse
White rhinoceros
Dog
Ferret
Panda
Megabat
Chinese pangolin
Microbat
Elephant
Manatee
Tenrec
Armadillo
Opossum
Tasmanian devil
Painted Turtle
Chicken
Turkey
Golden Eagle
Zebra Finch
Medium Ground Finch
Budgerigar
Brown kiwi
American Alligator
Anole lizard
Tibetan Frog
African clawed frog
Xenopus tropicalis
Coelacanth
B
LTR13
MER11A
MER65C
LTR10E
ZNF670
ZNF124
ZNF695
ZNF669
LOR1a
LOR1b
MER4D
MER72
HERV9-int
chr1.2
50kb
L1MEc
L1M4c
L1PA4
L1PA5
L1MC
L1MCa
L1Ms
L1M3
L1PA7
ZNF354B
ZNF354C
ZNF354A
ZNF454
ZNF879
MER11A
L1PB1
chr5.2
50kb
TE families
LINE/L1
LTR/ERVK
LTR/ERV1
LTR/ERVL-MaLR
target enrichment
least
most
enriched subfamilies

Figure S8: Evolutionary history of paralogs and additional examples of shared targets

A) Cross-species sequence comparison of ZFP69 (top) and ZFP69B (bottom). The alignment scores (see supplementary methods) between the zinc fingerprints of human ZFP69 or ZFP69B and all KZFPs of the indicated species are shown as boxplots. The dots corresponding to the best alignment score for each species are connected with a yellow line. Alignments were also done for the KRAB domain, but only the best alignments joined by a red line are shown. The number of identified potential non curated KZFPs (N), as well as the time of divergence (Age), for each species is indicated below. Silhouettes courtesy of http://phylopic.org/. B) Networks for clusters chr1.2 and chr5.2 were targets (circles) of each KZFP (squares) are shown as connected edges and the amount of binding is represented by the line thickness. The thickest line for each KZFP represents the TE subfamily with the highest $-\log_{10}$(FDR) and then scales linearly to the lowest value. For visibility, only the best targets (below) and shared targets (above) are shown. The TE subfamilies are colored according to their families. Primary targets for each KZFP are highlighted in red.

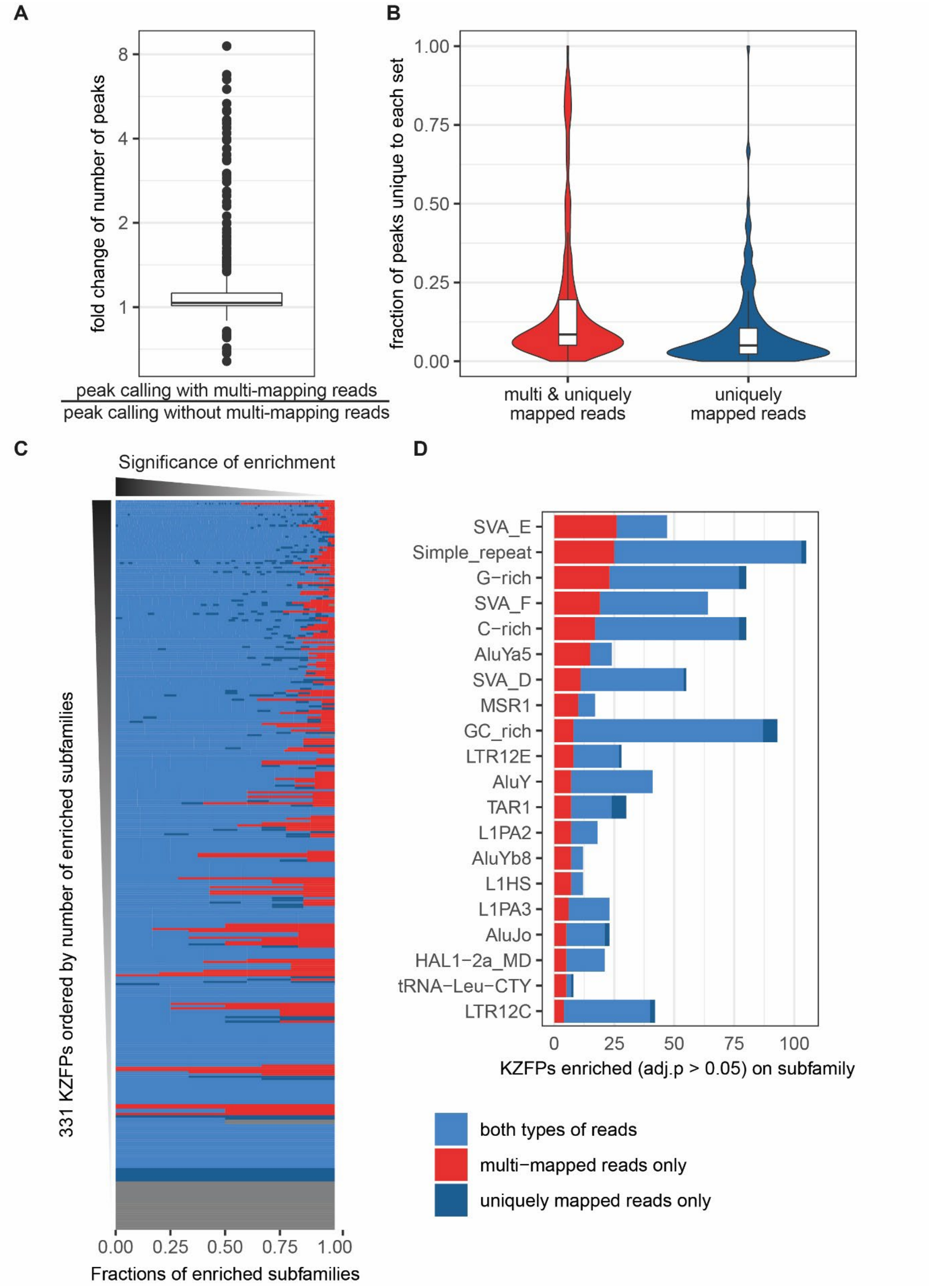
A
B
fold change of number of peaks
8
4
2
1
peak calling with multi-mapping reads
peak calling without multi-mapping reads
fraction of peaks unique to each set
1.00
0.75
0.50
0.25
0.00
multi & uniquely mapped reads
uniquely mapped reads
C
Significance of enrichment
331 KZFPs ordered by number of enriched subfamilies
0.00
0.25
0.50
0.75
1.00
Fractions of enriched subfamilies
D
SVA_E
Simple_repeat
G−rich
SVA_F
C−rich
AluYa5
SVA_D
MSR1
GC_rich
LTR12E
AluY
TAR1
L1PA2
AluYb8
L1HS
L1PA3
AluJo
HAL1−2a_MD
tRNA−Leu−CTY
LTR12C
0
25
50
75
100
KZFPs enriched (adj.p > 0.05) on subfamily
both types of reads
multi−mapped reads only
uniquely mapped reads only

Figure S9: Impact of multi-mapped reads on peak calling and target enrichment

A) Boxplot of the $\log_2$ transformed number of peaks obtained when including multi-mapped reads during peak-calling, divided by the number of peaks when excluding multi-mapped reads for each ChIP-seq experiment. The plot shows a slight increase of the total number of peaks when including multi-mapped reads in the majority of experiments. B) Violine plots showing the fractions of the total number of called peaks uniquely present when including multi-mapped reads (left, red) or when excluding them (right, blue) for all ChIP-seq experiments. This plot indicates the fraction of peaks that would be lost when excluding multi-mapped reads (left, red) or when including them (right, blue), showing that both adding and removing multi-mapped reads lead to a unique set of peaks for most experiments. C) Effect of multi-mapped reads on enrichment over Repeat Subfamilies. Each row in the plot represents the union of all significantly enriched subfamilies for one KZFP, both when including or excluding multi-mapped reads. The colored proportions show the fractions that were found to be enriched only when including multi-mapped reads in the analysis (red), only when excluding multi-mapped reads from the analysis (dark blue) or in both cases (light blue). KZFPs with no enriched subfamilies are shown in grey. KZFPs are ordered by the total number of enriched subfamilies with the highest on the top. Subfamilies for each KZFP are ordered by adjusted p-value, with the lowest p-values on the left. The plot shows that the Repeat subfamilies gained by including multi-mapped reads (red) are generally less significantly enriched than the ones gained by excluding multi-mapped reads (dark blue). D) Bar plot showing the twenty Repeat Subfamilies most positively affected by the inclusion of multi-mapped reads during the analysis. The bar width represents the number of KZFPs enriched on a subfamily. The colors indicated KZFPs enriched only when multi-mapped reads are included (red), only when multi-mapped reads are excluded (dark blue) or in both cases (light blue).

Supplementary Methods

Cross-species zinc fingerprint comparison

The cross-species comparisons of zinc fingerprints (ZFP) of KZFPs was built on the method described in (Imbeault et al. 2017). ZFPs were identified as described in the Methods section of this publication in the genomes of 69 different species including human (hg19, panPan2, panTro5, panTro4, gorGor4, ponAbe2, nomLeu3, chlSab2, colAng1, rhiBie1, macFas5, manLeu1, rhiRox1, papAnu2, macNem1, rheMac8, cerAty1, saiBol1, calJac3, aotNan1, otoGar3, proCoq1, micMur3, galVar1, criGriChoV1, cavPor3, dipOrd1, mm10, hetGla2, hetGla1, ochPri3, oryCun2, rn6, speTri2, vicPac1, manPen1, bosTau6, canFam3, turTru2, musFur1, equCab2, pteVam1, myoLuc2, balAcu1, ailMel1, susScr11, oviAri3, sorAra2, cerSim1, dasNov3, loxAfr3, triMan1, echTel2, monDom5, sarHar1, allMis1, anoCar2, aptMan1, melUnd1, galGal5, aquChr2, geoFor1, chrPic1, melGal5, taeGut2, xenLae2, nanPar1, xenTro9 and latCha1). In our analysis ZFPs were expanded to include the 4th DNA interacting amino acid (AA), located at position 2 of the alpha helix (Elrod-Erickson et al. 1998), as well as a special character "_" flanking each quartet of AAs from a individual zinc finger (ZF). The special character "_" was added to the Blosum80 matrix (Henikoff and Henikoff 1992) complemented with stop codons with a score of +100 against itself and -100 against any other character, in order to force alignments between entire ZFs. Each ZFP was then aligned globally against all other ZFPs with an opening gap penalty of -20, a gap extension penalty of 0 and no penalty for end gaps. To compare ZFP alignments, we developed a score named Complete Alignment of Zinc Finger (CAZF), which incorporates both the total number of matching amino acids as well as the number of perfectly matching ZF (all four AAs) between ZFPs. The rational for the inclusion of the latter part being that a ZFP with 4 different AAs in a single ZF (usually a single event like a deletion of an entire ZF) is more closely related to a reference ZFP than another ZFP which has 4 different AAs across 4 ZF (presumably 4 independent events). The score is normalized with the alignment of the ZFP with itself meaning that ZFPs with a score of 1 are identical.

$$CAZF\ score = \frac{1}{2}\left(\frac{AL_{score}}{AL_{self}} + \frac{ZF_{perfect}}{ZF_{self}}\right) \cdot \frac{Len_{seq}}{Len_{align}}$$

Where: AL_{score} is the alignment score using the modified BLOSUM80 matrix between the amino acids of the reference ZF and the target ZF, AL_{self} is the alignment of the reference ZF against itself (maximum score), $ZF_{perfect}$ is the number of ZFs that perfectly aligned (all four AAs), ZF_{total} the total number of ZFs in the reference sequence, Len_{seq} the length of the reference sequence, and Len_{align} the length of the alignment.

The last term was introduced to penalize short ZFP aligning to fractions of much longer ZFPs with high scores. Similarly, the KRAB domains are aligned and compared with the alignment against self:

$$KRAB\ score = \frac{AL_{score}}{AL_{self}} \cdot \frac{Len_{seq}}{Len_{align}}$$

Where: AL_{score} is the alignment score between the amino acids of the reference KRAB domain and the target KRAB domain, AL_{self} is the alignment of the reference KRAB domain against itself (maximum score), Len_{seq} the length of the reference sequence, and Len_{align} the length of the alignment.

Supplementary Tables (S1-S4: .xlsx files)

Table S1: Census of human KZFPs

Table S2: ChIP-seq data on human KZFPs

Table S3: Primary targets of KZFPs

Table S4: KZFP and TE age comparison

Supplementary References

Elrod-Erickson M, Benson TE, Pabo CO. 1998. High-resolution structures of variant Zif268-DNA complexes: implications for understanding zinc finger-DNA recognition. *Struct Lond Engl 1993* **6**: 451–464.

GTEx Consortium. 2017. Genetic effects on gene expression across human tissues. *Nature* **550**: 204–213.

Henikoff S, Henikoff JG. 1992. Amino acid substitution matrices from protein blocks. *Proc Natl Acad Sci U S A* **89**: 10915–10919.

Imbeault M, Helleboid P-Y, Trono D. 2017. KRAB zinc-finger proteins contribute to the evolution of gene regulatory networks. *Nature* **543**: 550–554.

Schmitges FW, Radovani E, Najafabadi HS, Barazandeh M, Campitelli LF, Yin Y, Jolma A, Zhong G, Guo H, Kanagalingam T, et al. 2016. Multiparameter functional diversity of human C2H2 zinc finger proteins. *Genome Res* **26**: 1742–1752.

Weirauch MT, Yang A, Albu M, Cote AG, Montenegro-Montero A, Drewe P, Najafabadi HS, Lambert SA, Mann I, Cook K, et al. 2014. Determination and inference of eukaryotic transcription factor sequence specificity. *Cell* **158**: 1431–1443.
